# Irisin‐Loaded Cerium‐Containing Mesoporous Bioactive Glass for Effective Immunomodulation and Odontogenesis of Dental Pulp Cells

**DOI:** 10.1002/advs.202501567

**Published:** 2025-07-11

**Authors:** Mingxin Wang, Yuxin Yang, Jingyao Guo, Yan Chen, Xiaolin Lu, Guangdong Zhang, Yuli Wang, Minhui Yao, Yue Liu, Qian Ma

**Affiliations:** ^1^ Department of General Dentistry The Affiliated Stomatological Hospital of Nanjing Medical University State Key Laboratory Cultivation Base of Research Prevention and Treatment for Oral Diseases Jiangsu Province Engineering Research Center of Stomatological Translational Medicine Nanjing Medical University Nanjing Jiangsu 210029 China; ^2^ State Key Laboratory of Digital Medical Engineering School of Biological Science and Medical Engineering Southeast University Nanjing 210096 China; ^3^ Department of Oral and Maxillofacial Surgery The Affiliated Stomatological Hospital of Nanjing Medical University State Key Laboratory Cultivation Base of Research Prevention and Treatment for Oral Diseases Jiangsu Province Engineering Research Center of Stomatological Translational Medicine Nanjing Medical University Nanjing Jiangsu 210029 China

**Keywords:** Ce‐MBGNs, dental pulp cells, odontogenesis, oxidative stress, vital pulp therapy

## Abstract

Recently, vital pulp therapy (VPT) has emerged as a promising strategy for dental pulp treatment because of the preservation of the pulp tissue. The efficacy of VPT relies on the use of VPT agents; however, commonly used VPT agents, including calcium hydroxide (CH), possess several limitations, such as dental pulp cells (DPCs) stimulation. In this study, we report the design of a novel VPT agent by loading Irisin onto cerium‐containing mesoporous bioactive glass nanoparticles (Ce‐MBGNs) to generate Irisin/Ce‐MBGNs. Compared to CH, which do not exhibit any obvious anti‐inflammatory effect, Irisin/Ce‐MBGNs have been shown to enhance the function of mitochondria in macrophages, regulate the M2 polarization, significantly reduce the intracellular ROS level, and alleviate the expression of pro‐inflammatory factors. Furthermore, the Irisin/Ce‐MBGNs nanocomposite effectively preserved DPCs stemness and promoted the odontogenic differentiation of human DPCs. Results in in vivo experiments showed that Irisin/Ce‐MBGNs could form thicker reparative dentin surrounding the perforation holes than CH. The superior treating efficacy of Irisin/Ce‐MBGNs is attributed to the synergistic effects of both Irisin and cerium components for the effective immunomodulation and odontogenesis of DPCs. Hopefully, this work contributes to the design of more advanced VPT agents for future implementation in clinical use.

## Graphical abstract

The Irisin/Ce‐MBGNs delivery system can slowly release myokine Irisin for vital pulp therapy. Irisin/Ce‐MBGNs can enhance mitochondrial function and promote the differentiation of inflammatory macrophages toward M2‐Mφ, thereby regulating the immune response mechanism of inflamed dental pulp. Irisin/Ce‐MBGNs also possess the ability to maintain cell stemness and effectively promote the odontogenic mineralization of dental pulp cells.

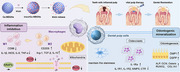

## 1 Introduction

According to a study in The Lancet in 2017 concerning the Global Burden of Disease (GBD),^[^ ^1^ ^]^ oral diseases ranked first among 354 diseases in terms of prevalence. Caries represents the most common form of oral disease, which could progress to cause pulpitis. Currently, the widely adopted treatment in the clinic for pulpitis is root canal treatment (RCT) for the complete removal of human pulp tissue, which could result in a complete loss of immune function and the possibility of re‐infection.^[^ ^2^ ^]^ Moreover, RCT‐treated the tooth after RCT usually more susceptible to fracture.^[^ ^3^, ^4^ ^]^ Consequently, it is desired to develop novel treatment technologies for pulpitis by preserving the pulp tissue because the dental pulp plays a crucial role in maintaining tooth vitality and protecting the tooth. For this purpose, vital pulp therapy (VPT), which aims to preserve the vitality and function of dental pulp tissue with minimal therapeutic intervention, has emerged as a promising alternative strategy for damaged pulp due to caries, trauma, or treatment.^[^ ^5^, ^6^ ^]^ The efficacy of VPT lies in the proper use of agents, including calcium hydroxide (CH) and mineral trioxide aggregate (MTA).^[^ ^7^ ^]^ However, these conventional VPT agents are facing several clinical challenges, including side effects of persistent chronic inflammation, pulp necrosis, tooth discoloration, and low dentin‐restorative capacity. Therefore, it is of a practical need to develop novel VPT agents to address the above issues and promote tissue regeneration in inflamed pulp tissues.

Pulpitis is closely associated with oxidative stress (OS).^[^ ^8^ ^]^ Aberrantly elevated levels of reactive oxygen species (ROS) can induce excessive OS that might result in cell death, the reduction of the osteogenic/odontogenic potential of dental pulp cells (DPCs), and the exacerbation of inflammation.^[^ ^9^, ^10^ ^]^ Therefore, control of the OS level in pulpitis is considered a promising strategy to promote pulp repair and regeneration.^[^ ^11^ ^]^ In such a context, Irisin has shown its potential to regulate OS levels by demonstrating both antioxidant and anti‐inflammatory properties.^[^ ^12^ ^]^ It also shows antioxidant activity counteracting the oxidative burst generated by macrophages in response to lipopolysaccharide (LPS) stimulation.^[^ ^13^, ^14^ ^]^ Furthermore, Irisin could also act as an excellent immunomodulator to regulate macrophage behavior by reducing excess ROS production.^[^ ^13^ ^]^ For example, previous studies have shown that exogenous Irisin could induce the polarization of adipose tissue macrophages from the M1 phenotype (M1‐Mφ) (pro‐inflammatory type) to the M2 phenotype (M2‐Mφ) (anti‐inflammatory type).^[^ ^15^ ^]^ In addition, the previous use of Irisin in periodontitis treatment has revealed its outstanding anti‐inflammatory properties,^[^ ^16^ ^]^ significantly reducing the macrophage‐mediated immune response in vitro, and its capacity to promote bone regeneration. Besides, Irisin has been shown to promote both odontogenic differentiation and angiogenesis in DPCs.^[^ ^17^ ^]^ The above features of Irisin imply that Irisin could be an ideal VPT agent upon proper use. It is important to note that Irisin is essentially a protein molecule; thus, the stability of exogenous Irisin in vivo remains a major challenge. In order to improve its stability and achieve its precise targeting and controlled release at inflamed sites, special delivery vehicles are required.

Nanocomposites have been widely used in the medical field for various advantages such as ease of preparation, functional versatility, low cost, and so on.^[^ ^18^, ^19^, ^20^ ^]^ Cerium‐containing mesoporous bioactive glass nanoparticles (Ce‐MBGNs) exhibit distinctive surface properties that can inhibit microbial growth and enhance tissue regeneration.^[^ ^21^ ^]^ Additionally, after the doping of cerium ions, Ce‐MBGNs could scavenge oxygen radicals and resist the early stage of pulp inflammation, resulting in an immune microenvironment conducive to the odontogenic differentiation of pulp cells.^[^ ^22^ ^]^ Degradation of Ce‐MBGNs releases bioactive ions (silica and calcium ions) that promote restorative dentin formation.^[^ ^23^ ^]^ Consequently, Ce‐MBGNs hold potential as VPT agents. Nevertheless, our recent study showed that the anti‐inflammatory property of Ce‐MBGNs is weak under concentrations that are optimized for tissue regeneration. Therefore, it is imperative to develop a rational design of Ce‐MBGN‐based VPT agents with enhanced anti‐inflammatory efficacy without compromising their regenerative effect. Ce‐MBGNs still retain a similar specific surface area similar to MBGN with a certain sustained release capacity. With the above knowledge acquired about Irisin, it is reasonable to hypothesize that a nanocomposite that integrates the functional of both Irisin and Ce‐MBGNs should achieve multiple goals, including the alleviation of OS in pulpitis, modulating inflammation, and thereby the promotion of the generation of reparative dentin.

In this study, we report the verification of our hypothesis by loading Irisin onto Ce‐MBGNs substrate to generate a nanocomposite Irisin/Ce‐MBGNs (Irisin‐loaded Cerium‐containing mesoporous bioactive glass nanoparticles). The obtained Irisin/Ce‐MBGNs were then used as a VPT agent for the treatment of pulpitis, marking the first use of Irisin for this purpose. In vitro assay, we evaluated the effects of Irisin/Ce‐MBGNs on the proliferation, migration, and anti‐inflammatory and odontogenic differentiation potential of DPCs. For the validation of the effect in vivo, an experimental model of rat pulpitis was constructed. Results confirmed that under inflammatory conditions, Irisin/Ce‐MBGNs reduced the expression level of pro‐inflammatory factors in RAW264.7 cells, as well as ROS in DPCs and RAW264.7 cells, and maintained the membrane potential of mitochondrial membranes. In addition, Irisin/Ce‐MBGNs could promote the proliferation of DPCs, improve the related RNA expressions of dentine regeneration, and also increase the activity of alkaline phosphatase (ALP). It was indicated that Irisin/Ce‐MBGNs promoted the odontogenic differentiation and mineralization of DPCs, which was further confirmed by the results of alizarin red, immunofluorescence, and western blot (WB) assays. In conclusion, it was demonstrated that Irisin/Ce‐MBGNs promoted dentin regeneration with good efficiency and achieved an improved anti‐inflammatory effect. Overall, this study not only suggests that Irisin/Ce‐MBGNs could be used as a potential VPT agent but also provides a novel protocol to develop advanced VPT agents for the clinical treatment of pulpitis.

## 2 Results

### 2.1 Physicochemical Characterization of Irisin/Ce‐MBGNs

**Figure 1:**
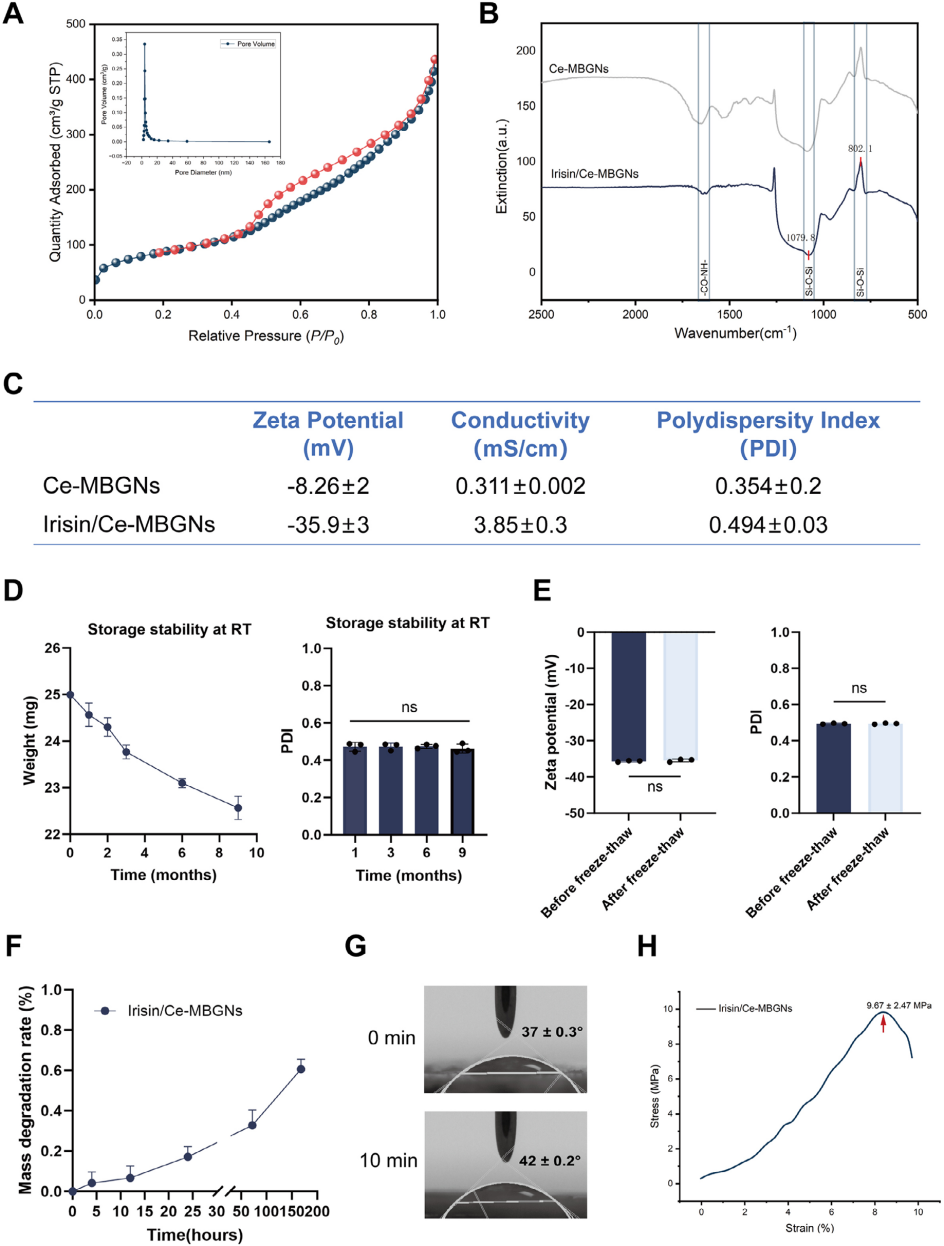
Physicochemical characterization of Irisin/Ce‐MBGNs. A) N_2_ adsorption‐desorption isotherms and inserted pore size distribution curves for Ce‐MBGNs. B) ATR‐FTIR spectra of Ce‐MBGNs and Irisin/Ce‐MBGNs. C) Zeta potential and PDI results (n = 3). D) Results of weighing measurements and Zeta potential tests were performed over a period of 9 months placing at room temperature (RT) (n = 3). E) Zeta potential of Irisin/Ce‐MBGNs after freeze‐thaw cycles for 5 times (n = 3). F) The mass degradation rate of Irisin/Ce‐MBGNs at 0, 4, 12, 24, 72, 168 h (n = 3). G) Contact angle measurement results before and after Irisin/Ce‐MBGNs were left at RT for 10 min. H) Compressive strength of Irisin/Ce‐MBGNs after 1 h (n = 30) (^*^ *p* < 0.05, ^**^ *p* < 0.01, ^***^ *p* < 0.001 and ns. *p* > 0.05. Values are expressed as mean ± SD).

The N_2_ adsorption‐desorption isotherm of Ce‐MBGNs conforms to the characteristics of a type IV isotherm, which is typical of mesoporous materials. What's more, the particles of Ce‐MBGNs maintained the narrow pore size distribution centered at 3.8 nm (**Figure** **1**A). The ATR‐FTIR spectra (Figure 1B) revealed characteristic silicate glass bands at 802.1 cm^−1^ (Si─O─Si bending) and 1079.8 cm^−1^ (Si─O─Si stretching)^[^ ^24^ ^]^ for Ce‐MBGNs and Irisin/Ce‐MBGNs. The presence of a distinct amide bond absorption peak in the range 1630–1680 cm^−1^ indicated the successful loading of Irisin onto the Ce‐MBGNs.

The zeta potential test results in pure water (Figure 1C) showed that both Ce‐MBGNs and Irisin/Ce‐MBGNs had negative surface charges. The absolute value of zeta potential between the nanoparticles of Irisin/Ce‐MBGNs increased, and the inter‐particle electrostatic repulsion also increased, indicating that the physical properties of the composite system were more stable than those of Ce‐MBGNs alone. The PDI of the nanoparticles increased slightly after loading Irisin, implying that the composite protein had little effect on particle distribution. The weighing measurement and zeta potential test results of Irisin/Ce‐MBGNs demonstrated that Irisin/Ce‐MBGNs could maintain good stability during long‐term storage at room temperature (Figure 1D). Besides, the freeze‐thaw cycle had no significant effect on the stability of Irisin/Ce‐MBGNs (Figure 1E). In vitro degradation experiments (Figure 1F) showed that Irisin/Ce‐MBGNs did not dissolve significantly in simulated body fluid and had good sealing performance. The contact angle of the Irisin/Ce‐MBGNs surface is less than 90 degrees, and it is hydrophilic (Figure 1G). After 10 min at room temperature, the contact angle increased slightly. The maximum compressive strength of Irisin/Ce‐MBGNs is ≈9.67 MPa within 1 h (Figure 1H), proving that Irisin/Ce‐MBGNs have sufficient mechanical strength.

**Figure 2:**
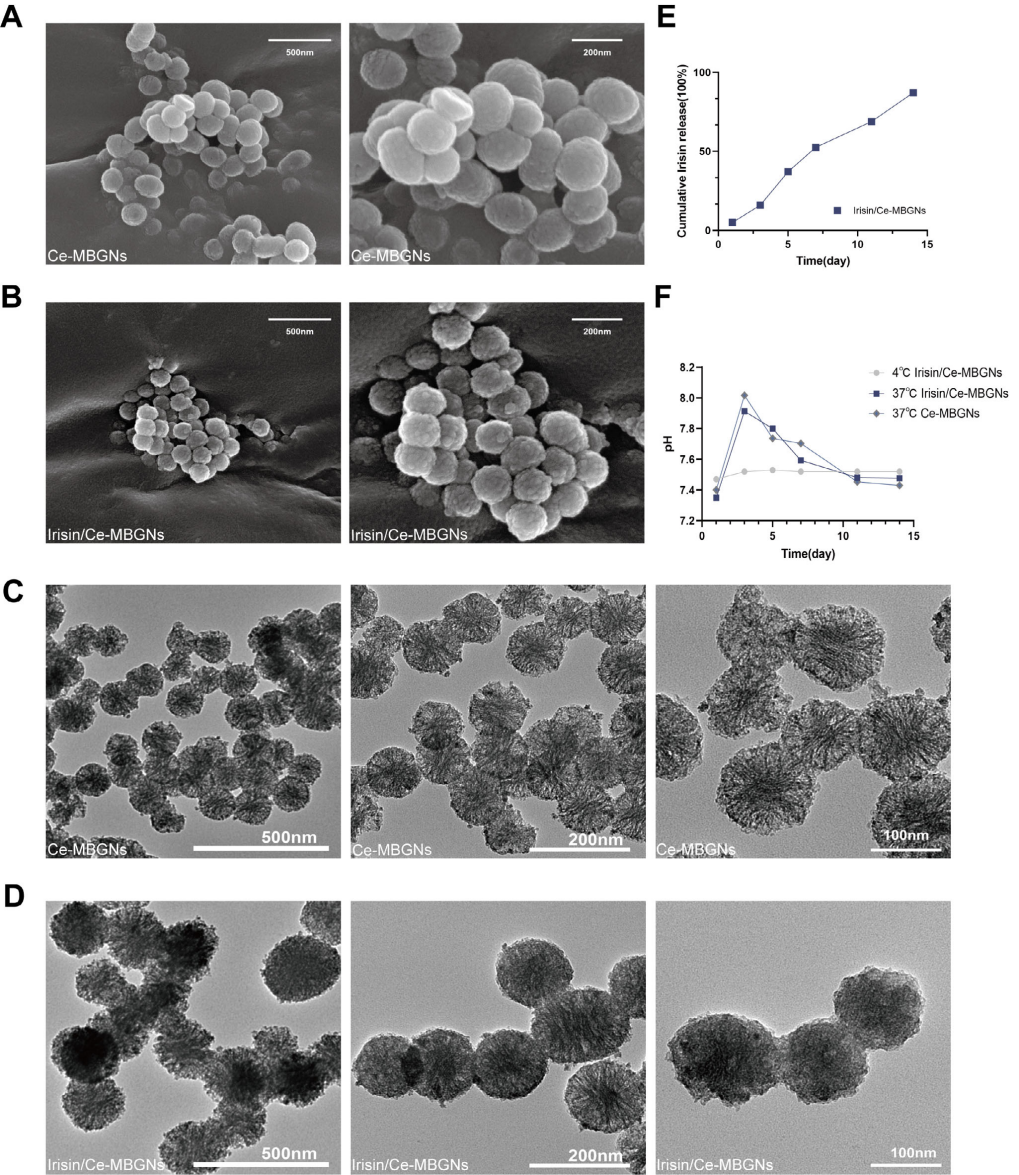
Synthetic of Ce‐MBGNs and Irisin/Ce‐MBGNs. A, B) SEM image of Ce‐MBGNs and Irisin/Ce‐MBGNs. C, D) TEM images of Ce‐MBGNs and Irisin/Ce‐MBGNs. E) Release of Irisin from Irisin loaded onto Ce‐MBGNs (Irisin/Ce‐MBGNs) (n = 3). F) Changes in the pH values of Ce‐MBGNs and Irisin/Ce‐MBGNs after immersion in SBF at 4 and 37 °C (n = 3).

In addition, the SEM results (**Figure** **2**A,B) revealed that Ce‐MBGNs were typical elliptic particles, with clear nanoparticles visible under a 200 nm scale under the microscope. Compared with the Ce‐MBGNs particles, Irisin/Ce‐MBGNs complex particles were rounder, the surface was rougher and protruding. Herein, the internal pores of the nanoparticles under a microscope at a 200 nm scale confirmed that Irisin had been successfully loaded. Moreover, the TEM results (Figure 2C,D) showed that compared with Ce‐MBGNs, Irisin/Ce‐MBGNs seemed to be more solid, replacing the heterogeneous pore with a cloudy complex center and a clearer boundary. It was speculated that Ce‐MBGNs particles had loaded enough Irisin.

The release profile of Irisin (Figure 2E) indicated that the Irisin loaded in Ce‐MBGN released ≈50% within the first 7 days, then the release rate slowed down and continued at a relatively stable rate. The result shows Irisin/Ce‐MBGN is a sustained‐release system that can act stably and effectively. The curve of pH value over time of Irisin/Ce‐MBGNs at 4 and 37 °C (Figure 2F) showed that Irisin/Ce‐MBGNs were stable at 4 °C and could be well preserved. At 37 °C, its pH value increased with the release of Irisin in the first 7 days, which gradually increased after the pH value reached the maximum and stabilized after day 14. The release of Irisin raises the pH value of Irisin/Ce‐MBGN in the prophase, but soon after the Irisin release rate decreases, and the pH of the complex changes to weakly alkaline and eventually remains stable.

### 2.2 Biocompatibility of Irisin/Ce‐MBGNs

**Figure 3:**
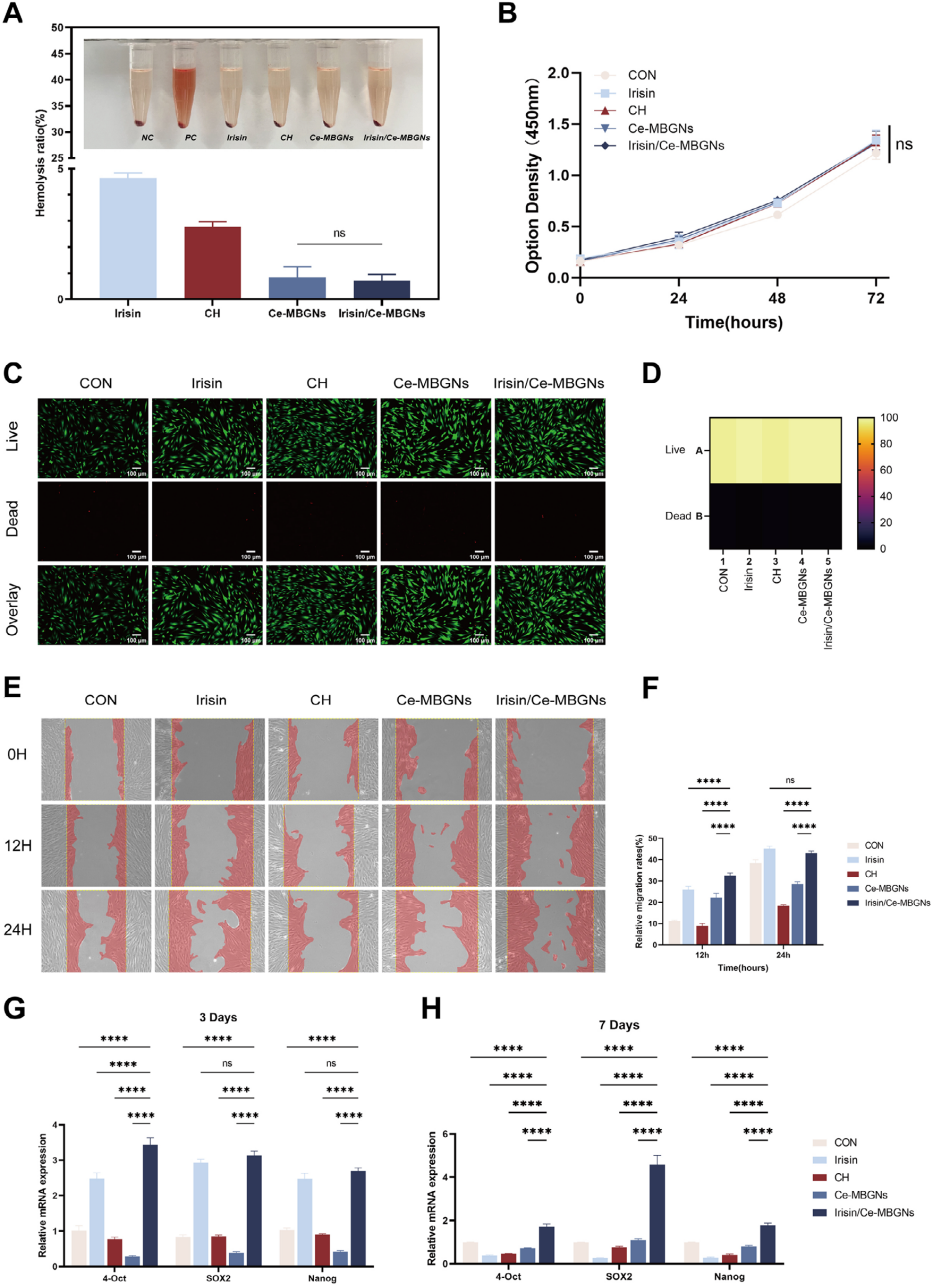
Biocompatibility of Irisin/Ce‐MBGNs. A) Hemolysis rate of materials (n = 3). B) CCK8 assay result of DPCs treated with Irisin/Ce‐MBGNs (n = 3). C) Representative fluorescence images and cluster heatmap (D) of live and dead cells. Live cells typically show bright green fluorescence, while dead cells show red fluorescence. E) Effects of Irisin/Ce‐MBGNs on cells migration of DPCs. F) Migration rate of Irisin/Ce‐MBGNs on DPCs (n = 3). G) Expression of stemness‐related mRNA (Oct‐4, SOX2, Nanog) at 3 days and 7 days (H) (n = 3). (^*^ *p* < 0.05, ^**^ *p* < 0.01, ^***^ *p* < 0.001 and ns. *p* > 0.05. Values are expressed as mean ± SD).

The hemolysis test results indicated that the hemolysis rates of Ce‐MBGNs and Irisin/Ce‐MBGNs were below 5% (**Figure** **3**A), meeting the threshold defined by medical device regulations. In order to test the biocompatibility of the prepared Irisin/Ce‐MBGNs, the results of CCK 8 were analyzed (Figure 3B), and all groups showed good biocompatibility within 72 h. This means that Irisin/Ce‐MBGNs can be safely used for biomedical applications. Live/dead staining results showed that the viability of all the cell groups was good (Figure 3C,D). The wound‐healing assay showed that Irisin/Ce‐MBGNs significantly accelerated the migration of DPCs, which was superior to Ce‐MBGNs after 4 and 24 h (Figure 3E,F). Thus, the addition of Irisin improved the cell migration ability of Ce‐MBGNs.

### 2.3 Maintenance Capability of Cellular Stemness of DPCs

The RT‐qPCR results (Figure 3G,H) showed that the expression levels of key transcription factors of stemness (Nanog, Sox2, Oct4) were significantly higher in the Irisin group and the Irisin/Ce‐MBGNs group on the third day. Admittedly, the expression of the stemness markers decreased in the DPCs after 7 days, but compared with the other groups, that of the Irisin/Ce‐MBGNs group was still high. That is to say, cells in the Irisin/Ce‐MBGNs group still had certain differentiation ability, and Irisin/Ce‐MBGNs could better maintain the stemness of the cells. On the contrary, after 7 days of co‐culture, the stemness of DPCs in the Irisin group decreased significantly and was even lower than that in the other groups. Its ability to maintain stemness was not long‐lasting, presumably because of the fast denaturation at 37 °C. After compounding with Ce‐MBGNs, Irisin could be obviously released slowly to achieve a more stable, prolonged, and excellent effect.

### 2.4 Irisin/Ce‐MBGNs Modulated Macrophage Phenotype and Reduced Cellular Oxidative Damage

**Figure 4:**
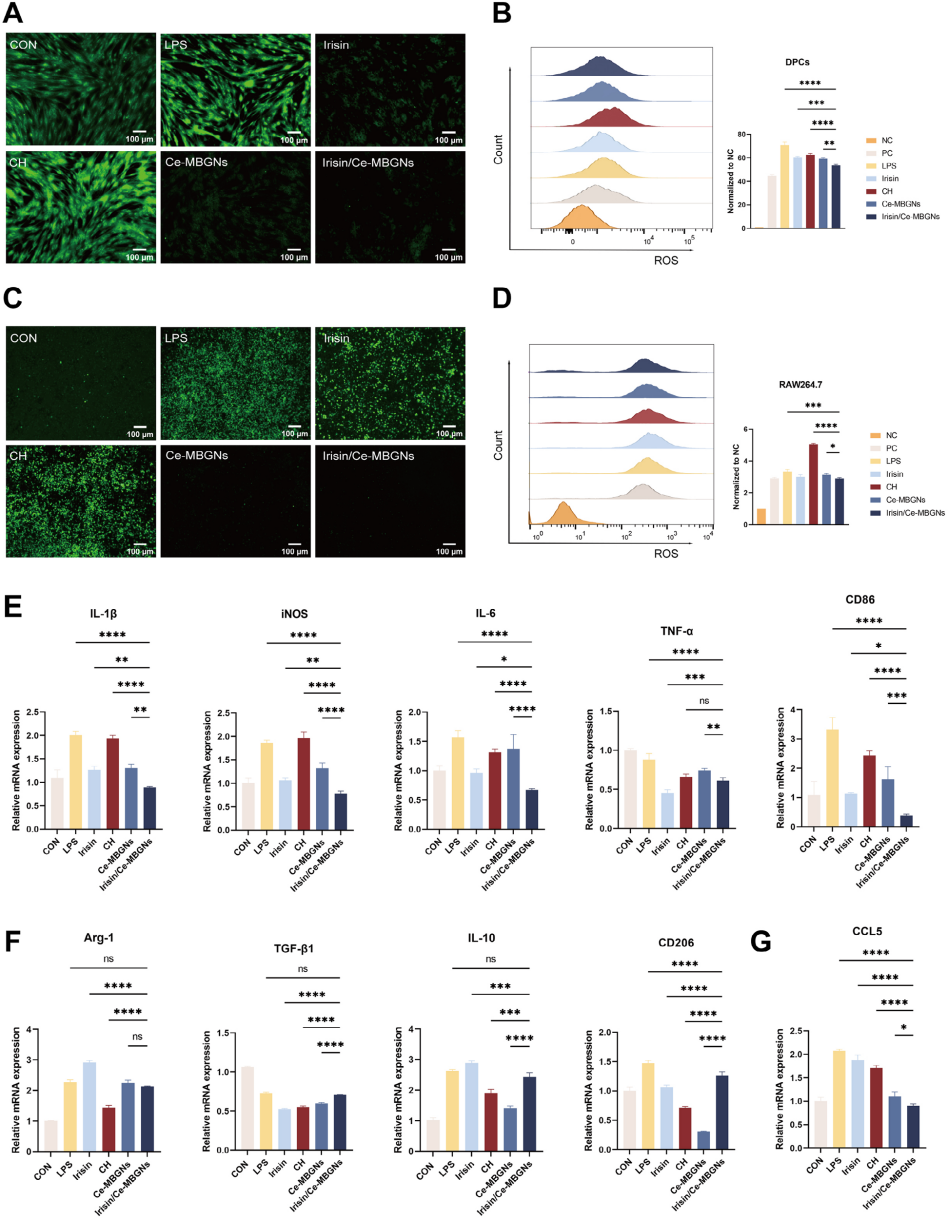
Irisin/Ce‐MBGNs reduce cellular oxidative damage and inhibit inflammation. A, C) Representative fluorescence images of DPCs and RAW. B, D) Intracellular ROS by flow cytometry (n = 3). E–G) RT‐qPCR quantitative analysis of the expression level of (E) M1 polarization‐related mRNA, (F) M2 polarization‐related mRNA, (G) CCL5 in RAW (n = 3). (^*^ *p* < 0.05, ^**^ *p* < 0.01, ^***^ *p* < 0.001 and ns. *p* > 0.05. Values are expressed as mean ± SD).

To explore the ability of the slow‐release system Irisin/Ce‐MBGNs to scavenge excess ROS within the inflammatory environment, inflammation was modeled in DPCs and RAW. The intensity of green fluorescence (FITC) in images taken by inverted fluorescence microscopy can indicate intracellular ROS levels (**Figure** **4**A,C). Irisin/Ce‐MBGNs notably reduced the fluorescence intensity in both cells. This indicated that Irisin/Ce‐MBGNs can effectively eliminate intracellular oxygen radicals, conferring resistance to endogenous oxidative stresses, and are more effective than Ce‐MBGNs alone. This shows that, on the basis of Ce‐MBGNs, Irisin/Ce‐MBGNs can further protect DPCs from self‐generated oxidative damage in an inflammatory environment. On the contrary, the fluorescence intensity of the CH group was similar to that of the LPS‐stimulated inflammation group, indicating that it has no obvious ability to scavenge ROS. At the same time, the ability of Ce‐MBGNs to scavenge ROS was quantitatively evaluated using flow cytometry. As shown in Figure 4B,D, the amount of fluorescence produced by the Irisin/Ce‐MBGNs group approximated that of the CON group without LPS treatment. Thus, it can be confirmed that Irisin/Ce‐MBGNs have excellent ROS scavenging ability.

**Figure 5:**
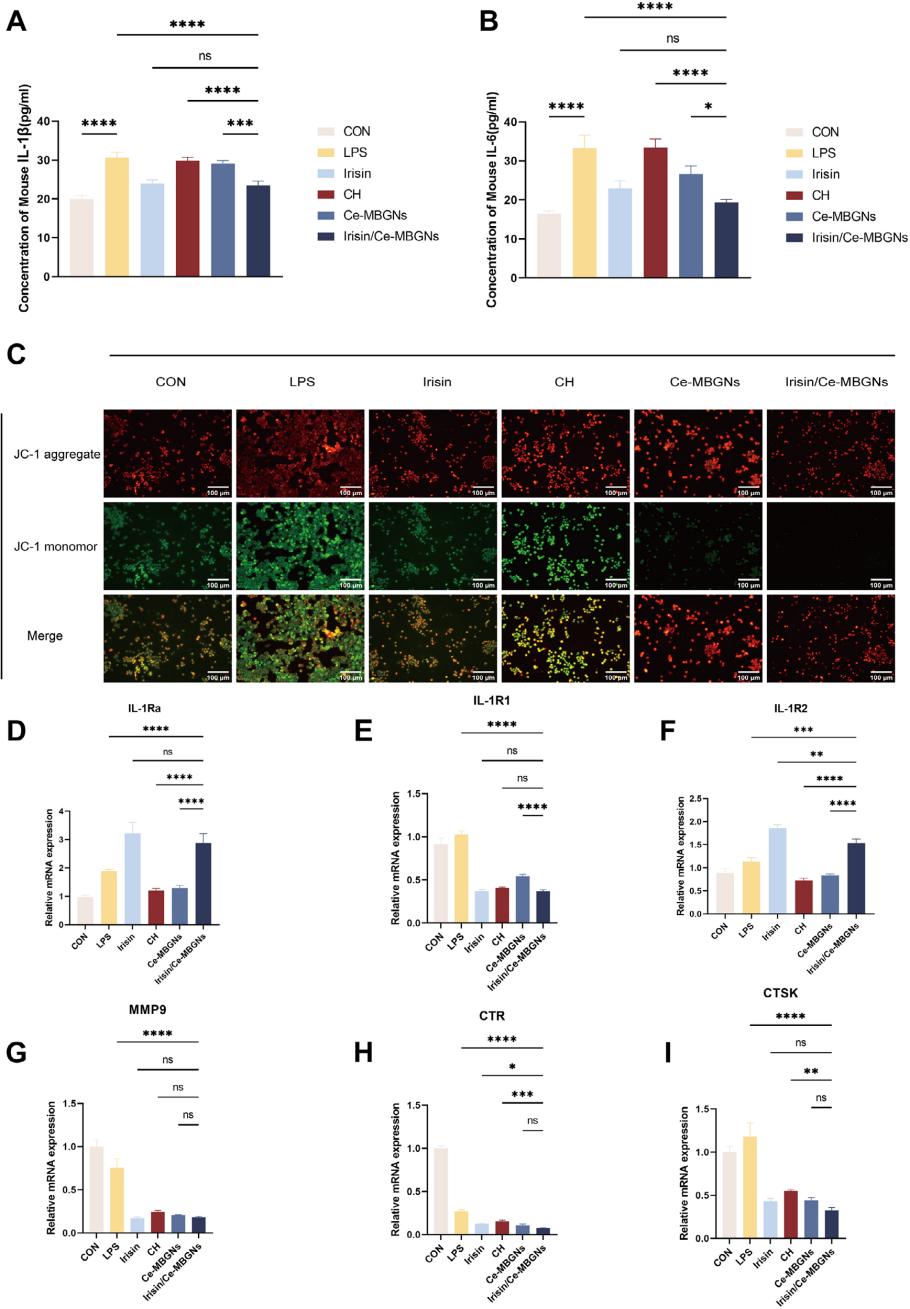
Irisin/Ce‐MBGNs reduce cellular oxidative damage and inhibit inflammation. A, B) Expression of IL‐1β and IL‐6 in the supernatant of RAW measured by ELISA (n = 3). C) MMPs of RAW by JC‐1 immunofluorescent staining. D–I) Expression of inflammation‐related and osteoclast differentiation‐related in DPCs after co‐culture with conditional medium for 24 h (n = 3). (^*^ *p* < 0.05, ^**^ *p* < 0.01, ^***^ *p* < 0.001 and ns. *p* > 0.05. Values are expressed as mean ± SD).

The RT‐qPCR results showed that the expression of M1‐Mφ markers (IL‐1β, iNOS, IL‐6, TNF‐α) in the Irisin/Ce‐MBGNs group was evidently reduced; especially, the expression of CD86 was almost 1/6 of that in the LPS group (Figure 4E). On the other hand, the Irisin group produced relatively more M2‐Mφ markers than CH and Ce‐MBGNs (Figure 4F), indicating that Irisin/Ce‐MBGNs also have the ability to regulate the phenotype of macrophages. The inhibitory effect of Irisin/Ce‐MBGNs on the chemokine CCL5 secretion was stronger than that of Ce‐MBGNs and Irisin (Figure 4G), indicating the ability of Irisin/Ce‐MBGNs to inhibit the infiltration and metastasis of inflammatory cells. What is more, the contents of IL‐1β and IL‐6, as the representatives of M1‐related pro‐inflammatory cytokines and inflammation‐associated factors present in the cell supernatants, were revealed to be decreased by ELISA (**Figure** **5**A,B). In short, Irisin/Ce‐MBGNs can promote the expression of anti‐inflammatory factors and inhibit the production of pro‐inflammatory factors, and their effect is superior to that of Ce‐MBGNs and Irisin used alone, and significantly better than that of the CH group.

To further explore the internal mechanism of immunomodulation, the MMPs of mitochondria in macrophages in the inflammatory environment were detected by immunofluorescence assay. The fluorescence characteristics of JC‐1 dye change with the change in the mitochondrial membrane potential: at high membrane potentials, it forms a red fluorescent polymer, while at low membrane potentials, it changes to a green fluorescent monomer. Accordingly, RAW was co‐cultured with CH, Ce‐MBGNs, and Irisin/Ce‐MBGNs for 24 h, then washed with PBS and co‐stained with JC‐1 dye. The immunofluorescence results (Figure 5C) showed that the Irisin/Ce‐MBGNs group had the least green fluorescent monomers, followed by the Ce‐MBGNs and Irisin groups. Relatively speaking, Irisin/Ce‐MBGNs had the most significant effect on the maintenance of MMP. Correspondingly, LPS, CH, and Irisin all experienced varying degrees of reduction of MMP and formed a larger number of green fluorescent monomers. These findings provide some evidence that Irisin/Ce‐MBGNs are able to enhance mitochondrial function in inflammatory macrophages via the preservation of MMP, achieving excellent immunomodulatory functions.

### 2.5 Irisin/Ce‐MBGNs Promoted Dentine Regeneration and Repair

Macrophage polarization plays a major role in inflammation and tissue regeneration. To confirm the effect of macrophages on the odontogenic/osteogenic differentiation of DPCs, RT‐qPCR was performed after culturing DPCs for 24 h using THP‐1 conditioned medium. The results showed that the expression of IL‐1Ra increased, whereas the expression of inflammation‐related and osteoclast differentiation‐related factors (IL‐1R1, IL‐1R2, MMP9, CTR) decreased (Figure 5D–I). These results suggested that Irisin/Ce‐MBGNs still had an inhibitory effect on osteoclasts in the inflammatory environment.

**Figure 6:**
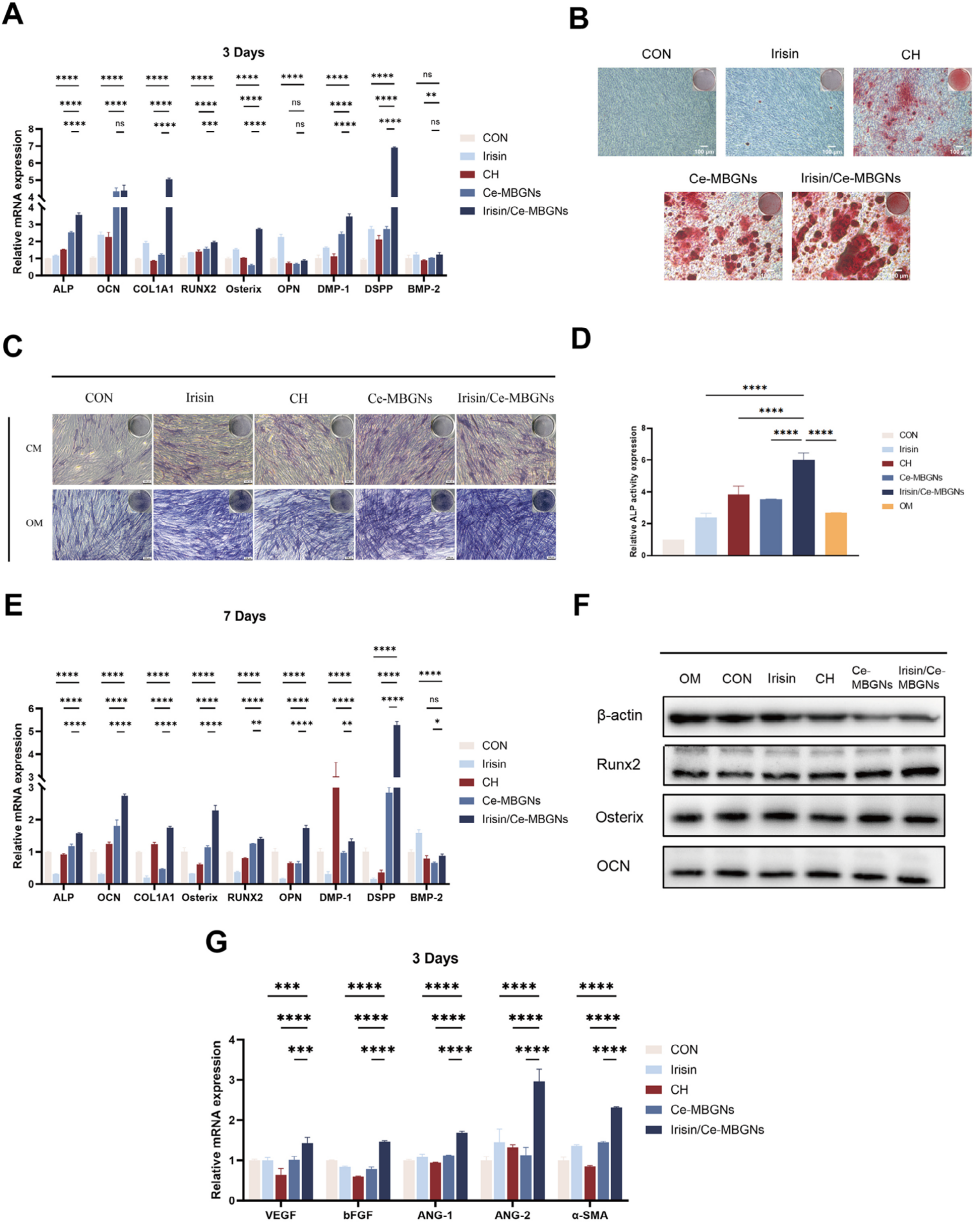
Effect of Irisin/Ce‐MBGNs on the promotion of dentine regeneration and repair. A) Dentine regeneration‐related gene expressions at 3 days (n = 3). B) Mineralization by alizarin red staining on day 21. C) Alkaline phosphatase (ALP) staining assay and ALP activity expression (D) of DPCs on day 7 (n = 3). E) Dentine regeneration‐related gene expressions at 7 days (n = 3). F) Osteo/dentinogenic differentiation‐related protein expressions of DPCs on day 7. G) Dentine regeneration‐related gene expressions at 7 days (n = 3). (^*^ *p* < 0.05, ^**^ *p* < 0.01, ^***^ *p* < 0.001 and ns. *p* > 0.05. Values are expressed as mean ± SD).

To further verify the role of Irisin/Ce‐MBGNs in promoting dentin regeneration, DPCs were co‐cultured with agents for 3 and 7 days. The expression levels of odontogenic/osteogenic differentiation‐related factors in each group of DPCs were compared by RT‐qPCR. Compared with Ce‐MBGN and Irisin stimulation alone, the mRNA expression levels of odontogenic differentiation markers (DSPP, DMP‐1, and BMP‐2) were significantly higher overall in the Irisin/Ce‐MBGNs group on day 3. The expression levels of osteogenic differentiation‐related factors (ALP, OCN, Osterix, RUNX2, and COL1A1) were significantly upregulated in the Irisin/Ce‐MBGNs group compared to the CH group (**Figure** **6**A). Admittedly, the expression levels of odontogenic/osteogenic differentiation‐related factors decreased in all groups at 7 days, but that of Irisin/Ce‐MBGNs still remained at a high level (Figure 6E). Even though the expression of OPN was highest in the CH group on day 3 (Figure 6A), it was gradually overstepped by the Irisin/Ce‐MBGNs group after 7 days (Figure 6E). From the above findings, it is obvious that Irisin/Ce‐MBGNs exhibit stable and long‐lasting pro‐dentin regeneration capacity. Furthermore, compared with CH, Irisin/Ce‐MBGNs showed excellent mineralization induction in DPCs (Figure 6C). The quantification of ALP activity also showed that the Irisin/Ce‐MBGNs group had the strongest ALP activity, which was superior to osteogenic induction solution alone and any other treatment (Figure 6D). Irisin/Ce‐MBGNs could enhance the effect of the osteogenic induction solution. Besides, after 21 days of incubation, the Irisin/Ce‐MBGNs group produced significantly more calcium nodules than the other groups (Figure 6B). At the protein level, we examined the expression levels of several important osteogenic markers (RUNX2, Osterix, OCN), which produced similar results (Figure 6F). The expression levels of vascular formation‐related markers (VEGF, bFGF, ANG‐1, ANG‐2, α‐SMA) were significantly higher than those in other groups, indicating that Irisin/Ce‐MBGN has the ability to support blood vessel formation (Figure 6G).

**Figure 7:**
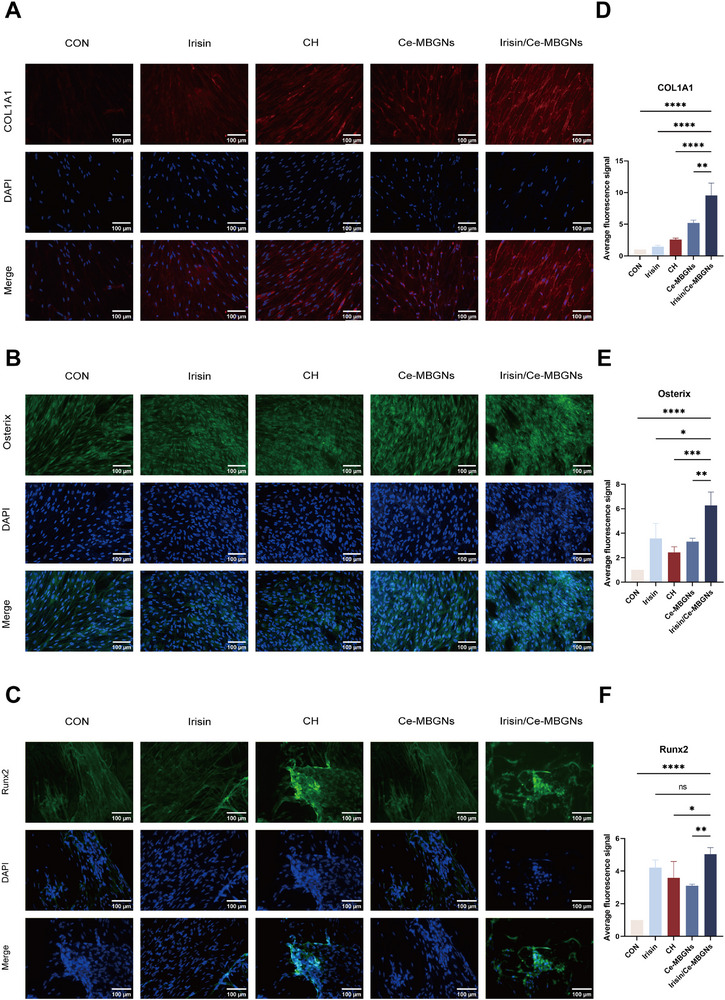
Irisin/Ce‐MBGNs accelerate the odontogenic differentiation of DPCs. A–C) Immunofluorescence staining of COL1A1, Osterix, and Runx2 in DPCs. D–F) Average fluorescence intensity of COL1A1, Osterix, and Runx2 in DPCs (n = 3). (^*^ *p* < 0.05, ^**^ *p* < 0.01, ^***^ *p* < 0.001 and ns. *p* > 0.05. Values are expressed as mean ± SD).

Apart from WB, the effects of Irisin/Ce‐MBGNs on the osteogenic differentiation of DPCs were examined using an immunofluorescence assay. Osteogenic‐related antibodies were used to de‐label proteins within DPCs, and the amount of protein expression within DPCs was reflected by the fluorescence intensity in the images captured by inverted fluorescence microscopy (**Figure** **7**A–C). Irisin/Ce‐MBGNs and Irisin alone enhanced nuclear translocation, and the Irisin/Ce‐MBGNs group enhanced the fluorescence signals of COL1A1, OPN, and RUNX2 to a greater extent than both single‐component groups (Figure 7D–F). In particular, stronger immunofluorescence was seen in the Irisin group than in the CH group and the CON group (osteoinductive medium group). These results suggest that Irisin/Ce‐MBGNs have the potential to contribute to the odontogenic/osteogenic differentiation and promote dentin regeneration and repair.

### 2.6 Dentin Repair Promoted by Irisin/Ce‐MBGNs in Rat Pulpitis

To verify the anti‐inflammatory and immunomodulatory effects of Irisin/Ce‐MBGNs in vivo, a model of pulpitis was established in the maxillary first molar of rats by 4 h of LPS stimulation. Irisin/Ce‐MBGNs or Ce‐MBGNs particles were used for VPT agents, and CH was used as a positive control (Figure S1, Supporting Information).

**Figure 8:**
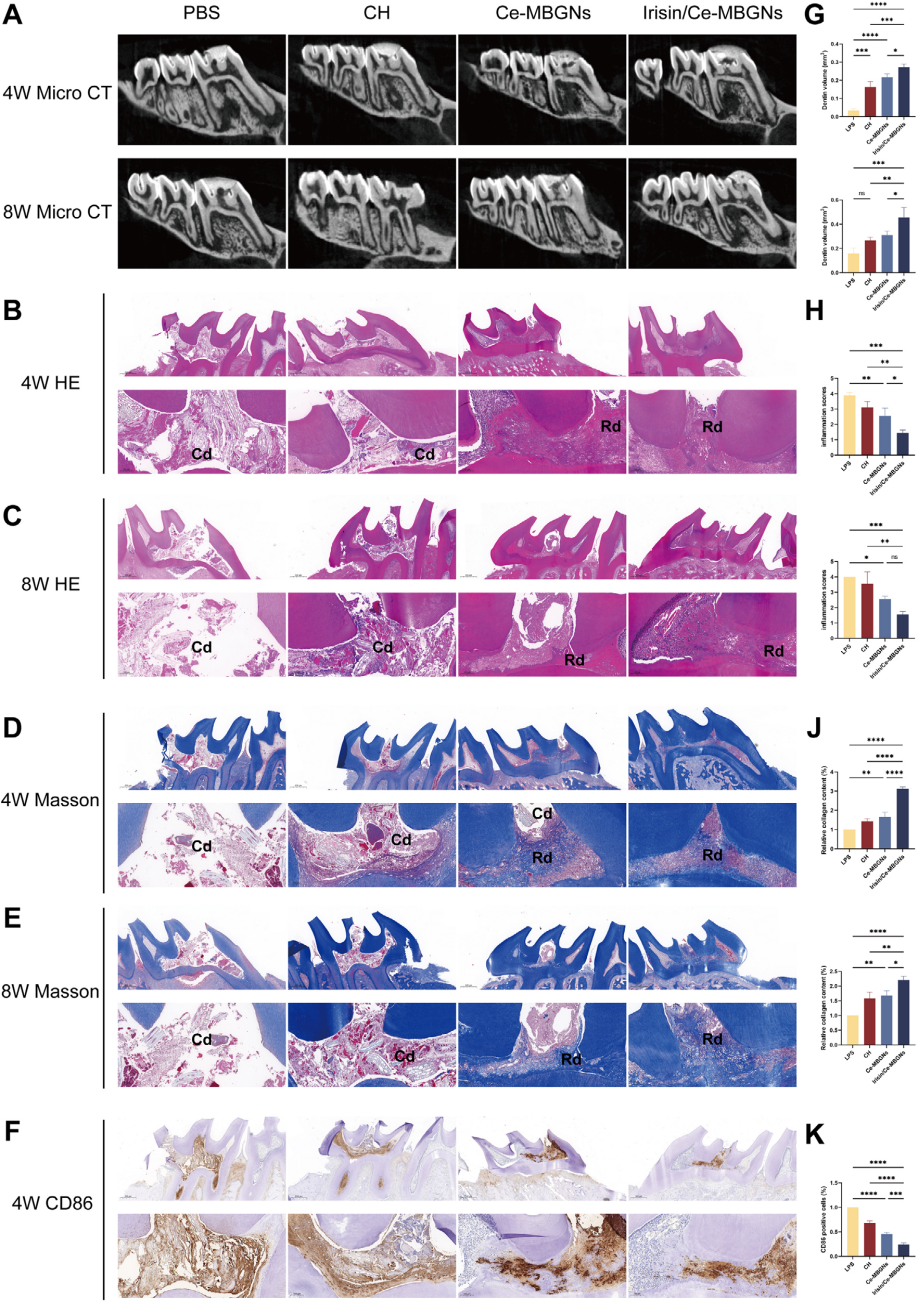
Dentin repair was promoted by Irisin/Ce‐MBGNs in rat pulpitis. A) The dentin repair of dental pulp in the rat first molar was determined by Vertical plane images of micro‐CT at 4 and 8 weeks. B) The inflammatory response of rat pulpitis was determined by HE staining at 4 and 8 weeks. C) Scale bars indicate 500 µm (top figure) and 100 µm (bottom figure). D) The dentin repair of rat pulpitis was determined by Masson's staining at 4 and 8 weeks. E) Scale bars indicate 500 µm (top figure) and 100 µm (bottom figure). F) CD86‐positive M1 macrophages were determined by IHC staining. Scale bars indicate 100 µm. G) Quantitative analysis of dentin volume at 4 and 8 weeks (n = 3). H) The semiquantitative analysis of the inflammatory response score (n = 3) at 4 and 8 weeks. J) The semiquantitative analysis of relative collagen (n = 3) at 4 and 8 weeks. K) The semiquantitative analysis of CD86‐positive cells at 4 weeks. (^*^ *p* < 0.05, ^**^ *p* < 0.01, ^***^ *p* < 0.001 and ns. *p* > 0.05. Values are expressed as mean ± SD).

After 4 and 8 weeks, micro‐CT was performed to assess the formation of restored dentin at the pulpal puncture site. The results (**Figure** **8**A) showed pulp necrosis in the vicinity of the pulpal perforation in the PBS group at 4 weeks, with a marked absence of surrounding dentin restoration. Scattered mineralization was observed within the pulp cavity in the CH group, and initial dentin bridging was seen near the pulpal perforation in the Ce‐MBGNs group. It is noteworthy that the mineralized bridges in the Irisin/Ce‐MBGNs group were clearer and thicker. After 8 weeks, the shadows near the pulpal perforation were further enlarged in the PBS group, while no significant changes were seen in the CH group, and a thin layer of mineralized tissue with ectopic mineralization in the pulp cavity was observed at the pulpal perforation in the Ce‐MBGNs group. Moreover, the Irisin/Ce‐MBGNs group had a clearer and stronger structure than previously. Quantitative analysis showed that more reparative dentin had been formed in the Irisin/Ce‐MBGNs group than in the Ce‐MBGNs group.

The biosafety of Irisin/Ce‐MBGNs was good, and no abnormalities were observed in vital organs such as the heart, liver, spleen, lungs, or kidneys (Figure S2, Supporting Information). The grade of inflammation and the formation of reparative dentin were assessed by HE staining analysis (Figure 8B,C). In the PBS group, the crown pulp was filled with fibrous connective tissue lacking cellular structure. Owing to the fact that the pulp self‐repair process was unable to eliminate the inflammation, most of the crown pulp tissue was disorganized and fissured after 8 weeks. The CH group exhibited disorganized calcified tissue in the area adjacent to the perforation hole, with localized formation of a large abscess in the remaining pulp. Besides, the restorative dentin formed close to the pulpal wall; after 28 days, its crown marrow tissue was completely disorganized and disintegrated. At 4 weeks, no obvious liquefaction occurred in the Ce‐MBGNs group, but a large local abscess formed near the perforation hole, with a clear inflammatory demarcation line visible around it. In addition, a local micro‐abscess appeared at 8 weeks, with an inflammatory demarcation line around it. In contrast, minimal infiltration of inflammatory cells was observed near the perforation of the pulp in the Irisin/Ce‐MBGNs group, and the layer of odontoblasts remained predominantly intact. After 8 weeks, there was no significant enlargement of the inflammatory extent of the coronal pulp in the Irisin/Ce‐MBGNs group, and odontoblast‐like cells were aligned underneath the reparative dentin. These results were further confirmed by Masson's trichrome staining (Figure 8D,E).

Through immunohistochemical (IHC) staining, the number of CD86‐positive M1‐Mφ was quantified in each group (Figure 8F,K). The PBS group and the CH group exhibited pulp necrosis and a significant increase in the number of CD86‐positive M1‐Mφ. In contrast, the Irisin/Ce‐MBGNs group showed a significant reduction in the area occupied by CD86‐positive M1‐Mφ, with a notable decrease compared to the Ce‐MBGNs group. The above results demonstrated that the Irisin/Ce‐MBGNs group could regulate the oxidative stress microenvironment induced by pulpitis, inhibit the progression of inflammation, and accelerate the tissue repair process, thereby facilitating the formation of reparative dentin.

### 2.7 Irisin/Ce‐MBGNs Regulated the Inflammatory Microenvironment and Promoted Dentin Repair Through Multiple Pathways

**Figure 9:**
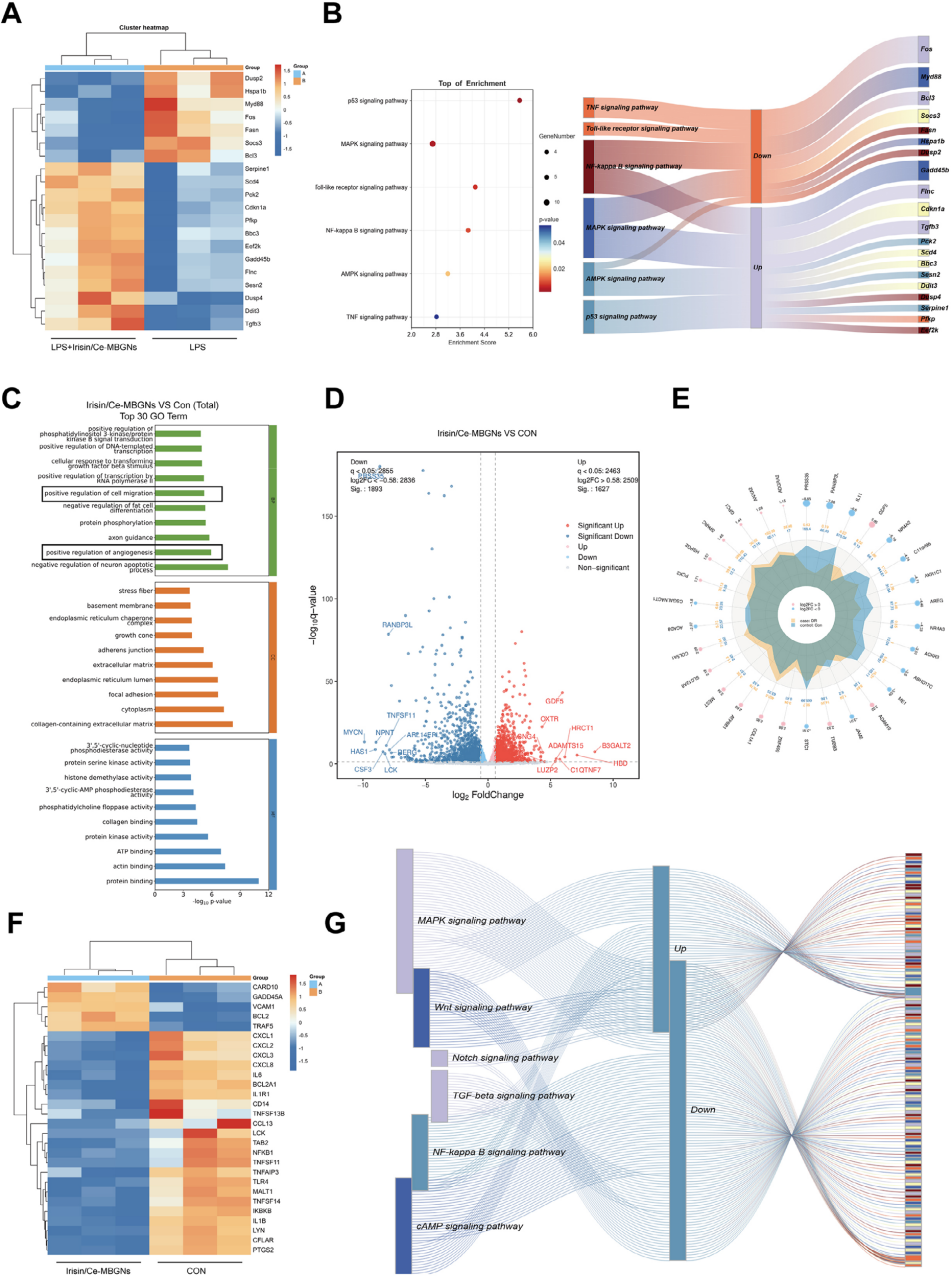
Irisin/Ce‐MBGNs regulated the inflammatory microenvironment through multiple pathways. A) Cluster heatmap analysis of the top 20 genes from transcriptome sequencing data for comparing between groups. For low and high expression levels, specifically, the colors blue and red were used. B) Significance of gene enrichment differences among pathways (bubble chart) and the connectivity between pathways (Sankey chart). C) Functional description of differentially expressed genes after GO enrichment analysis. D) Overall distribution of differentially expressed genes by volcano plot and radar chart (E). Red/pink indicates significant up, blue indicates significant down. The yellow spike corresponds to a high expression of a gene in the control group. F) Cluster heatmap analysis of differential genes from the NF‐κB pathway between groups. For low and high expression levels, specifically, the colors blue and red were used. G) The connectivity between several signaling pathways by a Sankey chart.

To further investigate the mechanism of action of Irisin/Ce‐MBGNs in pulpitis, RNA sequencing was used to analyze differentially expressed genes in the LPS and LPS + Irisin/Ce‐MBGNs groups. A cluster heatmap analysis was performed on the differentially expressed genes in the transcriptomic sequencing data, identifying 20 key genes from five distinct pathways, with overlapping and cascading interactions (**Figure** **9**A,B). The expression levels of a key adaptor protein in the Toll pathway (Myd88); a transcription factor (Fos); and an endogenous negative feedback molecule in the NF‐κB pathway (Bcl3), were downregulated compared to the LPS group, thereby inhibiting the TLR‐NF‐κB‐MAPK‐TNF cascade, suppressing the release of pro‐inflammatory factors, and rapidly inhibiting M1 polarization. Regarding the key negative regulatory factors of the MAPK signaling pathway, the overexpression of Dusp4 inhibited the duration of p38/JNK, while the downregulation of Dusp2 prevented insufficient early inflammatory inhibition. The increased expression of Gadd45b, Bbc3, Cdkn1a, Sesn2, and Serpine1 indicated the transcriptional activation of the p53 signaling pathway, coordinating immune clearance and tissue repair, and further inhibiting the activation of NF‐κB. Notably, increased expression of Sesn2 activated the AMPK signaling pathway, with downstream effectors of the AMPK pathway, while Scd4 and key genes (Pfkp/Pck2) showed increased expression. The above results, together with increased abundance of Tgfb3 in the MAPK pathway (Figure 9B), collectively facilitated the transition to the ‘metabolic‐repair’ phase.

In order to further explore the mechanism of Irisin/Ce‐MBGNs, genes with significant differences in DPCs were screened out. The functions of the differentially expressed genes between Con and Irisin/Ce‐MBGNs were described after GO enrichment analysis. The results showed that the differentially expressed genes were associated with positive regulation of angiogenesis, promotion of cell migration, and other functions (Figure 9C). The volcano plot and radar chart showed (Figure 9D,E) that the top/down 30 genes with the smallest q‐value or p‐value. Among these genes, the expression of TNFSF11 (i.e., RANKL), a factor related to osteoclast differentiation downstream of NF‐κB, was downregulated, which may reduce osteoclast activity and favour the formation of mineralized tissue. The downregulation of IKKβ expression in the NF‐κB pathway‐related differentially expressed genes (Figure 9F) indicated that the classical NF‐κB pathway was inhibited, and the expression of pro‐inflammatory factors IL1R1, IL6, and IL1β was downregulated. The fact that chemokines CCL13, CXCL1‐3, and CXCL8 were also downregulated further confirms that inflammatory responses were suppressed. Growth differentiation factor 5 (GDF5) promoted cell proliferation and differentiation by activating the Smad signaling pathway and then promoting mineralization matrix deposition. Additionally, RANBP3L is a nuclear export factor for BMP‐specific SMAD1/5/8, playing a key role in terminating BMP signaling and regulating mesenchymal stem cell differentiation. Its downregulation may indicate enhanced BMP signaling, which is beneficial for osteogenic/odontoblastic differentiation. The primary collagen component of dentin, COL1A1, was significantly upregulated (Figure 9E), suggesting activation of the Wnt/β‐catenin pathway. This pathway plays an important role in dentine formation. Downregulation of inhibitors of the Wnt signaling pathway (FRZB, NOTUM, and SFRP1) made it easier to activate Wnt signaling and thereby enhanced pathway activity. Upregulation of CACNG4 (calcium channel subunit) and OXTR (oxytocin receptor) may synergistically promote mineralization through calcium signaling and cAMP pathways (Figure 9G). In summary, Irisin/Ce‐MBGNs could inhibit inflammation‐related pathways, regulate the inflammatory microenvironment, and promote the activation of repair‐related pathways to facilitate dentin repair.

## 3 Discussion

Vital pulp therapy represents a promising alternative approach in endodontics aimed at maintaining pulp viability and function.^[^ ^25^ ^]^ Preserving and exploiting the pulp's innate immune defense mechanisms are key to successful VPT treatment.^[^ ^26^ ^]^ When the dental pulp is stimulated by inflammation, macrophages are among the most important immune cells attracted to the stimulated site. These cells originate from monocytes and reversibly polarize to M1 or M2 phenotypes according to appropriate immune stimulation. Importantly, the response of macrophages to the surrounding microenvironment determines whether they promote tissue repair or induce damage. Therefore, VPT agents that can ameliorate the inflammatory microenvironment and promote the self‐repair of pulpal tissues have a promising future in clinical applications. In this study, Irisin/Ce‐MBGNs were synthesized as potential agents for VPT. Irisin/Ce‐MBGNs could facilitate the self‐healing process of inflamed pulp by enhancing mitochondrial function and promoting macrophage M2 polarization. In our rat pulpitis model, Irisin/Ce‐MBGNs also exhibited significant anti‐inflammatory properties, immunomodulatory function, and the ability to accelerate reparative dentin formation.

Compared to Ce‐MBGNs, the PDI of Irisin/Ce‐MBGNs was increased (Figure 1C). Although Irisin increased the particle size, it also established a more stable composite system that allows long‐term storage at 4 °C. The physical and chemical properties of Irisin/Ce‐MBGNs were found to be more stable than those of the single‐component particles, and the effect is more lasting and effective. It is speculated that this is one of the reasons why the stemness of DPCs treated with Irisin/Ce‐MBGNs is better maintained. In addition, the absolute value of the zeta potential of Irisin/Ce‐MBGNs was augmented compared to Ce‐MBGNs (Figure 1C). To some extent, the increase in electrostatic repulsion between particles makes the Irisin/Ce‐MBGNs boundary clearer (Figure 2E,F). Negatively charged Irisin/Ce‐MBGNs can be retained in positively charged inflammatory sites by electrostatic adsorption, and the release of Irisin can be targeted in response to inflammation. Over and above that, it has been shown that the more negative charge the material surface carries, the better its effect on promoting osteogenic differentiation.^[^ ^27^ ^]^ Alterations in surface charge may potentially influence macrophage polarization, induce immunomodulatory effects, and promote osteogenic differentiation.^[^ ^28^ ^]^

Nanocomposites offer multiple advantages, including enhanced drug stability and solubility, sustained release, protection from degradation, and targeted delivery to the site of action.^[^ ^20^ ^]^ Therefore, Irisin/Ce‐MBGNs remained stable in long‐term storage at room temperature (Figure 1D,E). In a seven‐day in vitro degradation experiment, Irisin/Ce‐MBGNs showed no significant degradation (Figure 1F) and mainly exerted their function through bioactive ion release. This result also indicated that their solubility in oral fluid was minimal, which helped maintain the structural integrity of the material. Compared with biodegradable materials, their long‐term sealing properties were still relatively good.^[^ ^29^ ^]^ The sealing of restoration in VPT is another critical point for the clinical success of VPT treatment. Biological materials such as GeLMA, hydrogel, and chitosan exhibit swelling rate,^[^ ^30^ ^]^ which may disrupt the closure of the restoration. In contrast, the high hydrophilicity of Irisin/Ce‐MBGNs, similar to MTA, IRoot BP Plus, and Biodentine,^[^ ^31^ ^]^ ensures that it can still cure efficiently in a moist environment, enhancing marginal seal. One of the most important characteristics of VPT drugs is adequate compressive strength, which ensures that the material can withstand masticatory forces and contributes to the long‐term durability of the restoration.^[^ ^32^ ^]^ Ideally, the compressive strength of the repair material should be comparable to that of dentin or permanent restorative materials.^[^ ^33^ ^]^ The compressive strength of MTA is ≈4.50 MPa within an hour.^[^ ^32^ ^]^ The results of compression experiments showed that the compressive strength of Irisin/Ce‐MBGNs is ≈9.67 MPa (Figure 1H), indicating improved mechanical performance and potential for long‐term clinical application. MBGNs exhibit higher loading efficiency and slower release rate compared to traditional mesoporous SiO_2_ materials.^[^ ^34^, ^35^, ^36^, ^37^ ^]^ Doped with metal ions (e.g., Ag⁺, Zn^2^⁺, Sr^2^⁺) can give the material antimicrobial, pro‐angiogenic, or immunomodulatory functions.^[^ ^38^, ^39^, ^40^ ^]^ Irisin/Ce‐MBGNs are a mature, sustained‐release system that can stably and effectively release the loaded Irisin in a slow manner (Figure 1D). Although Ce‐MBGNs are believed to have certain immunomodulatory effects, the therapeutic effect of 1 mg mL^−1^ Ce‐MBGNs on inflammation was not as effective as we expected in a pro‐inflammatory environment. Therefore, the early release of Irisin could enhance the immunomodulation of Ce ions, effectively capture excessive ROS, and reprogram macrophage phenotypes. MBGN can release bioactive ions to promote tissue regeneration activities (such as bone formation, angiogenesis, etc).^[^ ^41^ ^]^ The release of metal ions from Ce‐MBGNs in the later stage could further promote dentin repair, and the antioxidant properties of Irisin and the mechanical support of Ce‐MBGNs could regulate the pulpitis microenvironment to promote tissue regeneration. Furthermore, the pH of Irisin/Ce‐MBGNs extract was in the range of ≈7.5–8.5 (Figure 2E), which is most favorable for tooth remineralization. Compared to CH, the stable nature of Irisin/Ce‐MBGNs reduced the irritation to the pulp and lowers the risk of pulp necrosis.

ROS are important mediators of inflammation, and the main source of intracellular ROS is mitochondria, especially in macrophages.^[^ ^42^ ^]^ When macrophages are activated by inflammatory stimuli, the metabolic activity of mitochondria is enhanced, generating excess ROS that damage the mitochondrial membrane by altering its permeability and reducing its MMP. The reduction of MMP disrupts the redox balance of mitochondria, further increasing the rate of ROS production, leading to a vicious cycle that ultimately damages the cell. The flow cytometry results showed that Irisin/Ce‐MBGNs could be targeted to reduce overexpressed ROS, which is consistent with the properties of Irisin itself. Meanwhile, the immunofluorescence staining of JC‐1 MMPs showed normal mitochondrial function in the Irisin/Ce‐MBGNs group and a significant decrease in mitochondrial membrane potential in LPS‐induced inflammatory macrophages, which indicated that mitochondrial function was impaired in the LPS group; this might also be related to mitochondrial senescence. In short, Irisin/Ce‐MBGNs protected MMP from harmful ROS‐induced depolarization in inflammatory environments, thereby preserving mitochondrial function, and are potent mitochondrial protectors. Mitochondria may be the main organelle where Irisin/Ce‐MBGNs exert their protective effects. In an inflammatory environment, mitochondria undergo a series of changes,^[^ ^43^ ^]^ including depolarization of MMPs, a metabolic shift from ATP synthesis to ROS production, and so on. These adaptive changes in mitochondria ultimately promote the production and release of pro‐inflammatory factors by activating the NF‐κB signaling pathway.^[^ ^44^ ^]^ Our findings provide some insights into the immunomodulatory mechanism of Irisin/Ce‐MBGNs.

ROS is also a marker of inflammatory macrophages (M1‐Mφ), which may be affected by macrophage polarization and may regulate the direction of macrophage polarization by affecting macrophage metabolic pathways. Macrophages change their phenotype during the inflammatory response and thus secrete various cytokines to counteract the associated stimuli.^[^ ^45^ ^]^ In the preliminary study, Irisin was shown to inhibit macrophage M1‐directed differentiation and promote M2‐polarization,^[^ ^46^ ^]^ which plays a key role in alleviating inflammation and promotes tissue regeneration.^[^ ^47^ ^]^ Pro‐inflammatory macrophages (M1‐Mφ) typically express IL‐1β, iNOS, IL‐6, and TNF‐α with surface markers CD86; correspondingly, anti‐inflammatory macrophages (M2‐Mφ) produce high levels of Arg‐1, TGF‐β, and IL‐10 with surface markers CD206.^[^ ^48^ ^]^ It was shown that Irisin/Ce‐MBGNs could significantly downregulate M1‐Mφ‐related factors, while M2‐Mφ‐related factors were rapidly upregulated (Figure 3E,F). Moreover, CCL5 plays a role in mediating the directed chemotaxis of immune cells and enhances the infiltration and metastasis of inflammatory cells. Macrophage chemokines are key molecules in regulating neutrophil recruitment during inflammation.^[^ ^49^ ^]^ Specifically, the chemokine CCL5 expressed in macrophages is critical in the immune response to pro‐inflammatory cytokines. Irisin/Ce‐MBGNs can effectively hinder the infiltration and spread of inflammatory cells by inhibiting the secretion of chemokine CCL5 (Figure 3G). The immunomodulatory ability of Irisin/Ce‐MBGNs may arise from the sustained release of Irisin and the scavenging of ROS by Ce ions. The coexistence and surface ratio of Ce^3+^/Ce^4+^ in Ce‐containing nanoparticles are key to their enzyme mimetic and antioxidant.^[^ ^50^, ^51^ ^]^ In addition, the alteration of the zeta potential on the surface of Irisin/Ce‐MBGNs after compounding may also result in an enhanced effect. The synergistic release of Ce ions and Irisin from Irisin/Ce‐MBGNs was also as expected to obtain a more stable long‐term effect. This suggested that Irisin/Ce‐MBGNs targeted the reduction in overexpressed ROS and the modulation of activated macrophage phenotype, thereby significantly reducing the inflammatory response and creating an anti‐inflammatory environment necessary for tissue repair and regeneration.

Macrophages can influence stem cells through various paracrine mechanisms^[^ ^47^ ^]^ and play a major role in inflammation and tissue regeneration. This study further investigated the possible effects arising from the interplay between DPCs and macrophages. We indirectly co‐cultured Irisin/Ce‐MBGNs with DPCs and macrophages in an inflammatory environment to mimic intercellular synergistic effects in vitro. Irisin/Ce‐MBGNs were found to reduce the production of pro‐inflammatory factors in the local immune milieu, thereby significantly inhibiting osteoclast differentiation of pulp cells (Figure 4D–I). The slow release of Irisin, followed by the degradation of Ce‐MBGNs, broke the vicious cycle of the inflammatory environment, elicited the odontogenic differentiation of DPCs toward differentiation, providing protection against enhanced dentin repair. Research has indicated that macrophages facilitate tissue repair through the secretion of diverse cytokines.^[^ ^52^ ^]^ Our results also reaffirmed that the transition of macrophages from M1 to M2 plays a crucial role in creating the favorable microenvironment required for pulp tissue regeneration.^[^ ^53^ ^]^

In addition, compared with Ce‐MBGNs alone, Irisin/Ce‐MBGNs performed better in promoting the odontogenic differentiation of DPCs. Previous studies have shown that Ce‐MBGNs at a concentration of 1 mg mL^−1^ promote odontogenic differentiation of DPCs.^[^ ^22^ ^]^To compare the ability of Irisin/Ce‐MBGNs with that of the single component, we verified the expression levels of the relevant factors by RT‐qPCR, a common method for the in vitro detection of dentinogenic cell differentiation in DPCs, followed by alizarin red staining, ALP staining, and immunofluorescence staining. Finally, we verified their dentinogenic properties once again at the protein level. In contrast to osteogenic differentiation, no genes or proteins specific to odontoblast differentiation have been identified, with the exception of DSPP and DMP‐1.^[^ ^54^ ^]^ But studies have demonstrated the significance of BMP‐2 in odontoblast differentiation as well as dentin secretion.^[^ ^55^ ^]^ Therefore, early indicators of bone regeneration^[^ ^56^ ^]^ (BMP2, ALP, RUNX2, COL1A1, Osterix, and OPN) were used as complementary methods to assess pulp cell differentiation and mineralization. Our results suggest that Irisin/Ce‐MBGNs possess the ability to continuously promote odontogenic differentiation of pulp cells. In the process of bone regeneration, Irisin has been proven to promote the differentiation of osteoblast lineage cells and effectively inhibit osteoclastogenesis.^[^ ^57^ ^]^ Ce‐MBGNs, on the other hand, trigger and enhance bone tissue regeneration mainly through their dissolution products (release of Si^4+^, Ca^2+^, P^5+^ ions and precipitation of biomaterials). The release of Ce ions can facilitate the formation of apatite layers.^[^ ^58^ ^]^ These two materials have different mechanisms of bone formation and can work synergistically with each other. To some extent, it explains why Irisin/Ce‐MBGNs exhibit superior ability in promoting odontogenic differentiation of DPCs compared to their two individual components.

Interestingly, we found that Irisin/Ce‐MBGNs appear to synergistically regulate inflammatory signals, metabolic reprogramming, and responses to oxidative stress. Irisin/Ce‐MBGNs can significantly remodel macrophage function, modulating the immune microenvironment, and thereby initiating the inflammatory repair process. Through transcriptomic sequencing, we identified the key mechanism by which Irisin/Ce‐MBGNs promote dentin repair in an inflammatory environment: multi‐pathway regulation of macrophage phenotype conversion. LPS stimulation leads to TLR4 activation of MyD88‐dependent and MyD88‐independent pathways, which induce the production of pro‐inflammatory chemokines and cytokines.^[^ ^59^, ^60^ ^]^ After LPS successfully induced an oxidative stress environment in pulpitis, the effects of Irisin/Ce‐MBGNs exhibited a clear hierarchical pattern. The upstream blockade of TLR4 (downregulation of Myd88/Fos) serves as the foundation (Figure 9A),^[^ ^61^ ^]^ metabolic reprogramming (activation of AMPK and upregulation of Scd4) provides energy support,^[^ ^62^ ^]^ while Bcl3/Socs3 downregulation relieves inhibition of the repair program.^[^ ^63^, ^64^ ^]^ This multi‐pathway synergistic mechanism holds greater application potential than single‐pathway anti‐inflammatory approaches. Irisin/Ce‐MBGNs may reduce macrophage M1 polarization by blocking the NF‐κB/MAPK inflammatory cascade. Ideally, p53‐mediated apoptosis (upregulation of Bbc3/Cdkn1a) first eliminates damaged M1 cells, followed by Tgfb3/Scd4‐driven M2 conversion, thereby controlling inflammation spread while avoiding premature fibrosis leading to pulp chamber closure. In clinical applications, the pulp microenvironment significantly limits the efficacy of VPT, while Irisin/Ce‐MBGNs' multi‐pathway regulation of the pulp microenvironment may partially enhance VPT success rates and reduce application limitations. This mechanism helps suppress irreversible inflammation and support hypoxic adaptation in pulpitis. However, it is worth noting that while Serpine1 overexpression may induce pulp calcification, no significant calcification was observed over the 8‐week animal experiment. In the future, we will carry out long‐term in vivo experiments to further explore the therapeutic effects of Irisin/Ce‐MBGNs.

Because of the non‐degradation of Irisin/Ce‐MBGNs, the long‐term effect of Irisin/Ce‐MBGNs must be further verified. We stimulated healthy dental pulp cells to simulate the drug action environment during indirect pulp cover treatment with VPT. The RNA sequencing results of DPCs indicate that the NF‐KB pathway is inhibited in DPCs after stimulation by Irisin/Ce‐MBGNs (Figure 9F), demonstrating the material's stable anti‐inflammatory effect. In the differential gene analysis of Irisin/Ce‐MBGNs, the most significant upregulation was observed in GDF5, which may be related to odontoblast differentiation (Figure 9D). Studies have shown that GDF5 promotes osteoblast differentiation through the Smad and p38MAPK signaling pathways and is involved in angiogenesis.^[^ ^65^, ^66^ ^]^ After stimulation by Irisin/Ce‐MBGNs, PRSS35 was significantly downregulated (Figure 9D). PRSS35 affects the extracellular matrix proteome, thereby limiting cell proliferation and participating in the fibrosis process.^[^ ^67^ ^]^ The downregulation of PRSS35 may be related to tissue repair and regeneration capabilities. The fate determination of DPSCs plays a crucial role in their future development, which in turn impacts the success of pulp coverage in clinical practice. The impact of VPT materials on odontoblasts is critical in treatment, affecting the success rate. Studies have shown that MTA materials can induce inflammation in healthy pulp without an inflammatory environment, potentially inhibiting mineralization,^[^ ^68^, ^69^, ^70^ ^]^ which could limit their clinical application.

As seen from the above results, our study further improved the performance of Ce‐MBGNs. It was successfully demonstrated that Irisin/Ce‐MBGNs have a positive effect on immunomodulation, which can protect DPCs from self‐oxidative damage, and odontoblast differentiation of DPCs in inflammatory states. Key findings and comparisons between Irisin/Ce‐MBGNs and other VPT agents have been listed in Table S4 (Supporting Information). To sum up, Irisin/Ce‐MBGNs provide broad clinical prospects for VPT agents. Although our study has investigated the multi‐pathway regulatory mechanisms of Irisin/Ce‐MBGNs, the spatio‐temporal relationships underlying the activation of these mechanisms still require further exploration for more precise clinical application. In addition, Irisin/Ce‐MBGNs are still in the preclinical stage and require the development of more convenient application forms for ease of use.

## 4 Conclusion

In this study, we successfully constructed an Irisin‐containing cerium nanoporous bioactive glass drug sustained release system (Irisin/Ce‐MBGNs) and investigated its potential as a VPT agent. The results suggested that Irisin/Ce‐MBGNs can preserve MMP to enhance mitochondrial function, affect the polarization of macrophages, and inhibit intracellular ROS production, thereby modulating the pulp immune response. Furthermore, Irisin/Ce‐MBGNs could modulate the combined action of macrophages on DPCs and reduce cell damage in the inflammatory environment, thereby promoting the odontogenic differentiation of DPCs. Irisin/Ce‐MBGNs were further validated to promote restorative dentin and anti‐inflammatory properties in vitro in a rat pulpitis model. Overall, this work provides a comprehensive experimental and analytical support of this novel Irisin/Ce‐MBGNs for VPT treatment in terms of immunity and regeneration.

## 5 Experimental Section

### Preparation of Irisin/Ce‐MBGNs

500 mg of Ce‐MBGNs powder was dissolved in 50 mL phosphate‐buffered saline (PBS), followed by 100 ng mL^−1^ Irisin (Adipogen, Liestal, Switzerland), vigorously shaken, and vortexed for 20 s. After a 24‐h incubation at room temperature (RT), Irisin/Ce‐MBGNs solution was frozen at 80 °C overnight, then lyophilized to powder and stored at 20 °C. The extracts (Irisin/Ce‐MBGNs) were stored at 4 °C prior to use.

### Preparation of CH

Based on the existing preparation methods,^[^ ^71^, ^72^, ^73^ ^]^ 300 mg of the CH powder (KESHI, Chengdu, China) was dissolved in 50 mL of ultrapure water, vigorously shaken, and vortexed for 20 s. After a 24‐h incubation at 37 °C, the CH solution was centrifuged at 2,000 rpm for 5 min before collecting the supernatant. The supernatant was diluted with medium to a concentration of 0.25 mg mL^−1^. Hydrochloric acid was added to neutralise the medium containing CH (0.25 mg mL^−1^) to pH 7.5. The mixture was stored at 4 °C until further use.

### Characterization of Irisin/Ce‐MBGNs

The microscopic morphology of the prepared Irisin/Ce‐MBGNs was characterized by a field emission scanning electron microscope (SEM; ZEISS, Oberkochen, Germany) and a transmission electron microscope (TEM; JEM‐2100F, Japan). The zeta potential of Ce‐MBGNs and Irisin/Ce‐MBGNs was measured at RT utilizing a Zetasizer Nano ZS instrument (Malvern Panalytical, China). Dynamic light scattering (DLS) was used to examine polydispersity index (PDI) at 25 °C, with each measurement conducted between a minimum of 10 and a maximum of 100 times. For all measurements, the samples were dispersed in ultrapure water at a concentration of 1 mg mL^−1^. The analysis was done in triplicate.

The storage stability of Irisin/Ce‐MBGNs was evaluated by weighing measurements and zeta potential test on the sample after 9 months’ storage at room temperature (RT). After freeze‐thaw cycles for 1, 3, and 5 times freeze‐thaw cycles, the zeta potential of Irisin/Ce‐MBGNs was measured.

### In Vitro Degradation Test

The Irisin/Ce‐MBGNs were immersed in 2 mL simulated body fluid (SBF), and samples were collected at 0, 4, 12, 24, 72, and 168 h. The samples were freeze‐dried into powder, and the sample mass W_d_ (0 h corresponds to W_d0_, and the weight of the samples at sequential time points corresponds to W_dn_) was measured. The following formula was used to calculate the mass degradation rate (DR_in vitro_):

(1) $$DR_{\mathrm{in}\;\mathrm{vitro}}=\frac{\mathrm{Wd}0-\mathrm{Wdn}}{\mathrm{Wd}0}\%$$

### Contact Angle Test

The contact angle of pure water on the surface of Irisin/Ce‐MBGNs was measured using the drop‐on‐dip method by a contact angle tester (POWERE, China). Irisin/Ce‐MBGNs powder and distilled water were mixed in a 3:2 ratio to prepare test specimens. Drops of liquid were placed on the solid sample, and the shape images of the droplets were captured using a high‐frequency camera. The contact angle was calculated using Image J.

### Compression Experiment

According to the ISO 9917‐1:2007 guidelines, Irisin/Ce‐MBGNs powder and distilled water were mixed in a 3:2 ratio to prepare thirty test specimens for clinical consistency. The mixture was poured into a cylindrical mold (4 mm in diameter and 4 mm in height), and the specimens were removed after 1 h. The electronic universal testing machine (Instron, USA) was used to carry out the test at a loading force speed of 1.0 mm min^−1^. In order to ensure the accuracy of the data, pay attention to resetting the force display value when the force sensor touches the sample lightly, and then carry out the test. When the material was broken under the force, the pressure drops sharply, and the machine stops automatically.

### The pH Changes and the Release of Irisin

To study the pH change of Irisin/Ce‐MBGNs and assess the release rate of Irisin from Irisin/Ce‐MBGNs, Irisin/Ce‐MBGNs powder was immersed in simulated body fluid (SBF) using a uniform suspension of 120 rpm at 37 °C. The pH values were measured at days 1, 3, 5, 7, 11, and 14 using a pH instrument, and the values were plotted over time. A volume of 100 µL solution was collected on days 1, 3, 5, 7, 11, and 14, and the cumulative release of Irisin was quantified three times using the Irisin ELISA kit (CSB‐eq027943HU).

### Cell Preparation and Culture

Dental pulp cells (DPCs) from patients aged 13–18 years were checked and approved by the Ethics Committee of Nanjing Medical University (PJ2022‐193‐001). Informed consent was provided by all patients and their guardians. The pulp tissues used for the extraction of pulp cells in this study were healthy and free of any lesions. DPCs were cultured in α‐MEM (Gibco, Australia) supplemented with 10% fetal bovine serum (FBS) and 1% penicillin/streptomycin (P/S, Gibco, Australia) at 37 °C in a humid atmosphere containing 5% CO_2_. The osteogenic differentiation medium used to induce osteogenesis was α‐MEM supplemented with 0.1 µm dexamethasone, 10 mm β‐glycerophosphate, and 50 µg mL^−1^ ascorbic acid (Sigma, USA).

The RAW264.7 cells (RAW) were sourced from a murine‐derived macrophage cell line provided by the Cell Bank (Stem Cell Bank, Chinese Academy of Sciences) and cultured in DMEM (Gibco, Australia) supplemented with 10% FBS and 1% P/S at 37 °C in a humid atmosphere containing 5% CO_2_.

### Cytotoxicity and Cell Attachment Test—Cell Counting Kit‐8 (CCK‐8) Assay

DPCs were seeded in 96‐well plates at 1 × 10^5^ cells/well. The cytotoxicity and effects of Irisin/Ce‐MBGNs on DPCs were evaluated by a CCK‐8 kit (Beyotime, China). The optical density was measured at 450 nm using a microtiter plate spectrophotometer (Molecular Devices, USA). The cell viability was calculated by the following equation:

(2) $$\mathrm{Cell}\;\mathrm{viability}=\frac{\left(\mathrm{Absorbance}\;\mathrm{of}\;\mathrm{sample}-\mathrm{Absorbance}\;\mathrm{of}\;\mathrm{blank}\right)}{\left(\mathrm{Absorbance}\;\mathrm{of}\;\mathrm{control}-\mathrm{Absorbance}\;\mathrm{of}\;\mathrm{blank}\right)}\%$$

### Cytotoxicity and Cell Attachment Test—Hemolysis Determination

Fresh rabbit red blood cells (rRBCs) (Sbjbio, China) were used in hemolytic experiments, and treated with PBS (negative control, NC), ultrapure water (positive control, PC), CH, Irisin, Ce‐MBGNs, and Irisin/Ce‐MBGNs. The incubation time was 1 h. After centrifuging at 3,000 rpm for 10 min, the photograph was taken for visual comparison. The absorbance at 540 nm was measured for the supernatant from each treatment using a multimode microplate reader (Tecan Spark, Switzerland). The percentage of hemolysis was expressed as:

(3) $$\mathrm{Hemolysis}=\frac{\left(\mathrm{Sample}-\mathrm{NC}\right)}{\left(\mathrm{PC}-\mathrm{NC}\right)}\%$$

### Cytotoxicity and Cell Attachment Test—Live/Dead Staining Assay

DPCs were seeded in 96‐well plates at 1 × 10^5^ cells/well. After co‐culturing for 48 h, the cytotoxicity of Irisin/Ce‐MBGNs on DPCs was evaluated by a Calcein/PI Live/Dead Viability/Cytotoxicity assay kit (Beyotime, China). The cells were then viewed under an inverted fluorescence microscope (Leica Microsystems, Mannheim, Germany). Measurements were analyzed using Image J software 10 (Tree Star, USA).

### Cytotoxicity and Cell Attachment Test—Wound‐Healing Assay

The migration ability of DPCs was assessed by the wound healing assay. First, DPCs were seeded in 6‐well plates. When the cell density reached 80% confluence, the cells were scraped with a disposable sterile 10 µL pipette tip to form two horizontal linear scratches. The cells were rinsed with PBS and incubated in serum‐free media containing Irisin, CH, Ce‐MBGNs, and Irisin/Ce‐MBGNs. Scratch images were obtained after 0, 12, and 24 h of incubation using an inverted microscope (Leica DM IL, Mannheim, Germany) at 4× magnification. Wound images were processed in Adobe Photoshop 2024. Wound closure was calculated using ImageJ software.

### The Expression of Stemness‐Related mRNA by Real‐Time Reverse Transcription Polymerase Chain Reaction (RT‐qPCR)

DPCs were seeded in 6‐well plates and co‐cultured with Ce‐MBGNs, Irisin/Ce‐MBGNs, and CH. Seven days after induction, the relative gene expression levels of stemness markers were measured by RT‐qPCR. The steps for total RNA extraction, the reverse transcription process, and RT‐qPCR testing are detailed in the Supporting Information. The primer sequences used are shown in Table S1 (Supporting Information). The results were normalized using glyceraldehyde‐3‐phosphate dehydrogenase (GAPDH) as the reference gene and subjected to the 2−ΔΔCt method. All relevant experiments were performed in triplicate.

### Indirect Co‐Culture of DPCs and THP‐1 Macrophages

THP‐1 cells (Procell, China), a human mononuclear cell line that could differentiate into macrophages, were used to investigate the immunomodulatory effects of Irisin/Ce‐MBGNs. THP‐1 cells were cultured in RPMI‐1640 medium (Gibco, Australia) supplemented with 10% FBS and 1% P/S at 37 °C in a humid atmosphere containing 5% CO_2_. The differentiation of THP‐1 cells into macrophages was stimulated with serum‐free medium containing phorbol 12‐myristate 13‐acetate (100 ng mL^−1^) (PMA, Sigma–Aldrich).

After a 24‐h incubation, THP‐1 macrophages were subsequently cultured for 24 h in medium containing 100 ng mL^−1^ lipopolysaccharide (LPS, E. coli) and 20 ng mL^−1^ IFN‐γ to trigger their activation into M1‐Mφ. M1‐Mφ were co‐cultured with Irisin/Ce‐MBGNs, Ce‐MBGNs, Irisin, and CH; the positive control group of M1‐Mφ with common medium was called the LPS group, and the negative control group without LPS treatment was the CON group. After collection and centrifugation at 1000 rpm for 5 min, six sets of supernatants were then taken as the conditioned medium for use in indirect co‐culture experiments. Then taken as the conditioned medium for use in indirect co‐culture experiments. Each well of DPCs was evenly seeded into 6‐well plates. The expression of inflammatory and osteoclast‐associated mRNA factors was measured by RT‐qPCR after 24 h. The relevant experiments were performed in triplicate.

### Intracellular ROS of RAW and DPCs

After paving RAW and DPCs, 100 ng mL^−1^ of LPS was added and allowed to act for 24 h. Then, cells in the inflammatory state were co‐cultured with Irisin, Ce‐MBGNs, Irisin/Ce‐MBGNs for 24 h, and CH, called the Irisin group, Ce‐MBGNs group, Irisin/Ce‐MBGNs group, and CH group, respectively. The positive control group was cultured with ordinary medium after LPS induction, and the negative control (CON) group received no LPS treatment. Subsequently, 5 µm H2DCFDA (MCE, China) diluted with serum‐free medium was added. After a 30‐min incubation at 37 °C, images were acquired using an inverted fluorescence microscope (Leica Microsystems, Mannheim, Germany) with the same parameters for each sample.

In order to further quantitatively verify the effect of Irisin/Ce‐MBGNs on macrophages and dental pulp cells, the same method as the ROS clearance assay was used to add 5 µm H2DCFDA diluted with serum‐free medium at 37 °C for 30 min under light. After incubation, cells were gently scraped down with a cell scraper, resuspended in 100 µL PBS solution, and tested by flow cytometer (FACSAiraIISORP, BD Biosciences, USA). FlowJo Software, version 10 (Tree Star, USA), was used to analyze all the data.

### The expression of Inflammation‐Related mRNA by RT‐qPCR

Each well of DPCs was evenly seeded into 6‐well plates, LPS (100 ng mL^−1^) was added and allowed to act for 24 h, then co‐cultured with Ce‐MBGNs, Irisin/Ce‐MBGNs, and CH. The level of inflammation‐related gene expression was measured by RT‐qPCR. The primer sequences used are shown in Table S2 (Supporting Information). All relevant experiments were performed in triplicate.

### The Secretion of IL‐1β and IL‐6 by RAW by ELISA

The supernatants from each experimental group in the above experiments were collected and centrifuged at 1000 rpm for 5 min, in order to test the secretion of IL‐1β and IL‐6. The steps for ELISA are detailed in the Supporting Information.

### Mitochondrial Membrane Potential (MMP) of RAW

The MMP values of RAW were determined through immunofluorescence by the Mitochondrial membrane potential assay kit with JC‐1 (Beyotime, Shanghai, China). RAW were washed several times with serum‐free medium, followed by a 20‐min incubation at 37 °C with the working solution of JC‐1 dye. Subsequently, RAW were washed several times with JC‐1 dilution buffer. Images were acquired immediately using an inverted fluorescence microscope (Leica Microsystems, Mannheim, Germany) with the same parameters for each sample.

### Odontoblast Differentiation and Mineralization of DPCs

DPCs were evenly seeded into 6‐well plates and co‐cultured with Ce‐MBGNs, Irisin/Ce‐MBGNs, and CH. Seven days later, the relative gene expression levels of stemness markers were measured by RT‐qPCR. The primer sequences used are shown in Table S1 (Supporting Information). All relevant experiments were performed in triplicate.

DPCs were evenly seeded into 6‐well plates, and when the cell density reached 80%, the original medium was replaced with Ce‐MBGNs or Irisin/Ce‐MBGNs with or without osteogenic induction medium, and compared with the blank control groups with or without osteogenic induction medium. Seven days later, cells were stained by ALP staining using the BCIP/NBT Alkaline Phosphatase Color Development Kit (Beyotime, China) following the instructions for use provided by the manufacturer. Images were collected using an OfficeJet Pro L7580 scanner (HP, Palo Alto, CA, USA). ALP activity was assessed by measuring the specific enzymatic activities that determine the osteogenic potential of DPCs. The Alkaline Phosphatase Assay Kit (Beyotime, China) was used to measure the ALP activity. The absorbance at 405 nm was determined by using a spectrometer.

DPCs were evenly seeded into 6‐well plates and co‐cultured with Ce‐MBGNs or Irisin/Ce‐MBGNs with osteogenic induction medium. After 21 days of osteogenic induction, the cells were stained with alizarin red solution (LEAGENE, China). After washing out the excess dye, photos were taken using an OfficeJet ProL7580 scanner (HP, Palo Alto, CA, USA).

After protein extraction, the protein concentration was determined using Brandford protein assay. Following blocking, the membranes were treated with primary antibodies (β‐actin antibody, Runx2 antibody, Osterix antibody, and OCN antibody) and secondary antibodies (goat anti‐mouse or goat anti‐rabbit). The WB assay was detailed in the Supporting Information. Protein bands were captured by the LI‐COR scanner from Odyssey, with the aid of the SuperSignal West Femto reagent manufactured by Thermo Scientific.

DPCs were evenly seeded into 6‐well plates and co‐cultured with Ce‐MBGNs, Irisin/Ce‐MBGNs, and CH. Three days later, the relative gene expression levels of angiogenesis‐related genes were measured by RT‐qPCR. The primer sequences used are shown in Table S1 (Supporting Information). All relevant experiments were performed in triplicate.

### Immunofluorescent Staining

The experimental groups were divided into the blank control group, Irisin group, Ce‐MBGNs group, Irisin/Ce‐MBGNs group, and CH group. Seven days after adding the materials, cells were washed three times with PBS and then fixed with 4% paraformaldehyde for 15 min. Following blocking, the membranes were treated with rabbit COL1A1 antibody (Cell Signaling Technology, USA), rabbit OCN antibody (Cell Signaling Technology, USA), and rabbit RUNX2 antibody (Abbkine, USA). After thorough washing, DAPI (Thermo Fisher Scientific, Germany) was applied for 5 min at RT, followed by a 1‐h incubation with fluorescent secondary antibodies. Images were captured by a CCD camera (Leica DFC420C) and examined under a fluorescence microscope (Leica DMI3000B, Germany).

### Construction of Pulpitis in Rat Maxillary First Molars

The animal testing was approved by the local ethics committee (IACUC‐2310006). Forty male rats were randomly and equally assigned to each experimental group, with five animals per group to ensure adequate statistical power. After the pulp‐penetration surgery was completed (the assay was detailed in the Supporting Information), the exposed pulp area was covered with CH or Ce‐MBGNs or Irisin/Ce‐MBGNs, then filled with glass ions. Throughout the experimental cycle, soft food was provided to prevent shedding of the glass ions. The rats were checked every three days, and any loss of filling was promptly addressed by refilling to maintain consistent treatment effects. The experimental groups were divided into a) negative control group (sterile PBS), b) positive control group (CH), c) Ce‐MBGNs group, and d) Irisin/Ce‐MBGNs group. The rats were sacrificed at weeks 4 and 8 after surgery. The maxilla containing the first molar was rapidly isolated and immediately placed in 4% paraformaldehyde. In vitro fixation was continued for 48 h.

### Micro‐CT, Immunohistochemistry (IHC), and Histology Analysis

Four weeks after surgery, micro‐CT images were analyzed with software such as Mimics Reconstruction and CTAn 1.8. The visualization of the rat maxillary first molar was digitally reconstructed to show the reparative dentin (RepD). The quantitative analysis of RepD was performed by converting the CT value to dentine volume (mm^3^).

After collection, the maxillary alveolar bones were placed in 4% paraformaldehyde for fixation, then underwent decalcification, and ultimately sectioned into 4 µm‐thick slices. Hematoxylin‐eosin (HE) staining and Masson's trichrome staining were used to assess the degree of inflammatory infiltration and mineralization in the pulp tissue. These samples were stained with CD86 antibody (1:200) for IHC evaluation. Six regions were randomly selected from the region of interest (ROI) of each animal sample at a magnification of 200× for the quantification of positive areas. Image J software was used to calculate the percentage of the positive area.

### RNA Sequencing—RNA Isolation and Library Preparation

The total RNA of DPCs and RAW264.7 from the CON/LPS group, (LPS+) Irisin/Ce‐MBGNs group, was extracted using the Trizol reagent (Invitrogen, CA, USA) according to the manufacturer's protocol. RNA purity and quantification were evaluated using the NanoDrop 2000 spectrophotometer (Thermo Scientific, USA). RNA integrity was assessed using the Agilent 2100 Bioanalyzer (Agilent Technologies, Santa Clara, CA, USA). Then, the libraries were constructed using the VAHTS Universal V6 RNA‐seq Library Prep Kit according to the manufacturer's instructions. The transcriptome sequencing and analysis were conducted by OE Biotech Co., Ltd. (Shanghai, China).

### RNA Sequencing—RNA Sequencing and Differentially Expressed Genes Analysis

The libraries were sequenced on an Illumina Novaseq 6000 platform, and 150 bp paired‐end reads were generated. Raw reads of fastq format were first processed using fastp, and the low‐quality reads were removed to obtain the clean reads for subsequent analysis. The clean reads were mapped to the reference genome using HISAT2. FPKM of each gene was calculated, and the read counts of each gene were obtained by HTSeq‐count4. PCA analysis was performed using R (v 3.2.0) to evaluate the biological duplication of samples. Differential expression analysis was performed using DESeq2. Q value < 0.05 and foldchange > 2 or foldchange < 0.5 were set as the threshold for significantly differentially expressed genes (DEGs). The radar chart of the top 30 genes was drawn to show the expression of up‐regulated or down‐regulated DEGs using the R packet ggradar. Based on the hypergeometric distribution, GO, KEGG pathway, Reactome, and WikiPathways enrichment analysis of DEGs were performed to screen the significantly enriched terms using R (v 3.2.0), respectively. R (v 3.2.0) was used to draw the volcano plot, cluster heatmap, sankey chart, and bubble chart of the significant enrichment term.

### Statistical Analysis

Results were analyzed by using GraphPad Prism 9.0 and 10.0 software. All data were expressed as mean ± standard deviation (SD). The statistical sample sizes for each experiment were three unless otherwise indicated. For multiple comparisons, statistical significance was analyzed using one‐way analysis of variance (ANOVA), followed by Tukey's post hoc test, which was used when comparing all the conditions. Statistical significance was expressed as ^*^ *p* < 0.05, ^**^ *p* < 0.01, ^***^ *p* < 0.001, ^****^ *p* < 0.0001, and ns. *p* > 0.05.

## Conflict of Interest

The authors declare no conflict of interest.

## Author Contributions

Q.M. did conceptualization, investigation, methodology, review and editing, supervision, resources, project administration, funding acquisition, visualization. M.W. did investigation, methodology, wrote the original draft, formal analysis, data curation, validation. Y.Y. did review and editing, formal analysis. J.G. did review and editing. Y.C. did review and editing. X.L. did visualization. G.Z. did validation. M.Y. did software. Y.L. did methodology. Y.W. did data curation. All authors have read and agreed to the published version of the manuscript.

## Supporting information



Supporting Information

## Data Availability

The data that support the findings of this study are available from the corresponding author upon reasonable request.
