# Supplementary material for: Irisin‐Loaded Cerium‐Containing Mesoporous Bioactive Glass for Effective Immunomodulation and Odontogenesis of Dental Pulp Cells
Source: Adv Sci (Weinh). 2025 Jul 11;12(38):e01567. doi: 10.1002/advs.202501567 (PMC12520508; doi:10.1002/advs.202501567)
Supplement: Supplementary file 1 — Supporting Information [file ADVS-12-e01567-s001.docx]

Supporting Information

**Irisin-loaded Cerium-containing mesoporous bioactive glass for effective immunomodulation and odontogenesis of dental pulp cells**

*Mingxin Wang^a^, Yuxin Yang^a^, Jingyao Guo^a^, Yan Chen^a^, Xiaolin Lu^b^, Guangdong Zhang^a^, Yuli Wang^c^, Minhui Yao^a^, Yue Liu^a^, and Qian Ma^a^**

^a.^ Department of General Dentistry, The Affiliated Stomatological Hospital of Nanjing Medical University; State Key Laboratory Cultivation Base of Research, Prevention and Treatment for Oral Diseases; Jiangsu Province Engineering Research Center of Stomatological Translational Medicine; Nanjing Medical University. Nanjing 210029, Jiangsu, China.

^b.^ State Key Laboratory of Digital Medical Engineering, School of Biological Science and Medical Engineering, Southeast University, Nanjing 210096, China.

^c.^ Department of Oral and Maxillofacial Surgery, The Affiliated Stomatological Hospital of Nanjing Medical University; State Key Laboratory Cultivation Base of Research, Prevention and Treatment for Oral Diseases; Jiangsu Province Engineering Research Center of Stomatological Translational Medicine; Nanjing Medical University. Nanjing 210029, Jiangsu, China.

Corresponding Author:

Qian Ma, maqian@njmu.edu.cn

*Materials and methods*

*Preparation of MBGNs*

MBGNs were synthesized using a microemulsion-assisted sol-gel method. Briefly, 0.7 g of cetrimonium was dissolved in 33mL of deionized water, 10 mL of ethyl acetate and 7.0 mL of aqueous ammonia were added under stirring. Then, 3.6 mL of tetraethyl orthosilicate and 2.28 g of calcium nitrate tetrahydrate were added sequentially. The produced nanoparticles were first collected under centrifugation, then washed with deionized water and ethanol, and finally calcined in a furnace at 700 °C for 3 hours(h).

*Cell extraction and culture*

The pulp tissues were collected from patients aged 13 to 18 years old with the review and approval of the Ethics Committee of Nanjing Medical University (PJ2022-193-001). Informed consent was provided by all participants or their guardians. The pulp was removed from the pulp cavity and washed three times with PBS solution containing 2% penicillin/streptomycin (P/S, Gibco, Australia), then cut into small pieces of tissue, washed thoroughly and cultured in α-MEM (Gibco, Australia) supplemented with 20% FBS at 37 ℃ in 5% CO_2_ in a humid atmosphere.

*Real-time reverse transcription polymerase chain reaction (Real-Time RT-PCR, RT-qPCR)*

Total RNA was extracted and purified by the RNAeasy™ animal RNA extraction kit (centrifugal column) (Beyotime, Shanghai, China), then reverse transcribed using the PrimeScript RT Master Mix. The results were normalized using glyceraldehyde-3-phosphate dehydrogenase (GAPDH) as the reference gene and subjected to the 2^−ΔΔ Ct^ method. All relevant experiments were performed in triplicate.

*Enzyme-linked immunosorbent assay (ELISA)*

After being planted at the density of 3 × 10^5^ cells in each well in 6-well culture plates, the RAW264.7 cells were permitted to culture for full 24 h. After paving, 100 ng/mL of LPS was added and allowed to act for 24 h. Then, cells were co-cultured with Irisin, Ce-MBGNs, Irisin/Ce-MBGNs and CH for 24 h. Finally, the supernatants from each experimental group were collected and centrifuged at at 16 000g for 5 minutes, in order to test the secretion of IL-1β and TNF-α. Multi Sciences kits for ELISA were performed according to the instructions offered by the manufacturer.

*Western blot (WB)*

The proteins were extracted using the M-PER mammalian protein extraction reagent provided by Thermal Sciences. Protein concentration was determined using the Brandford protein assay (Beyotime). Each set of 20 mg of protein was separated by 10% SDS-PAGE and then placed onto a PVDF (polyvinylidene difluoride) membrane. Membranes were blocked with 5% skim milk at room temperature (RT) and then incubated with β-actin antibody (1:15 000), Runx2 antibody (1:1 000), Osterix antibody (1:1 000) and OCN antibody (1:1000) for 12 hours at 4 ℃. Thereafter, the membrane was treated with secondary goat anti-mouse (1:10 000; proteintech) or goat anti-rabbit (1:8 000; proteintech) for 1 h at room temperature.

*Construction of pulpitis in rat maxillary first molars*

With reference to the pulpitis model in maxillary first molars of rat^[1, 2]^, all SPF rats (males, 7 weeks old, 200 ~ 300 g) were allocated to the trial group at random. After rats were anesthetized with 1% barbiturate sodium, they were fixed on the operating table in the supine position, washed alternately with 3% hydrogen peroxide and saline, and the mouth and surrounding tissues were disinfected with 0.5% iodophor. Under water cooling conditions, No. 1/4 ball was used to drill the fovea at the occlusal surface of the first molar of the right upper jaw until the bottom of the socket was pink. Subsequently, the 15# K file/DG16 probe was gently penetrated, and the diameter of the myeloid hole was about 0.35 mm, with the slight bleeding point as the standard for successful perforation. The socket was washed with normal saline and excess water was removed with sterile small cotton balls. The exposed pulp area was covered by CH or Ce-MBGNs or Irisin/Ce-MBGNs, then filled by glass ions. The experimental groups were divided into (a) negative control group (sterile PBS), (b) positive control group (CH), (c) Ce-MBGNs group, and (d) Irisin/Ce-MBGNs group. Both mandibular first molars were appropriately lowered, and the whole experiment was performed in strict accordance with aseptic procedures. The contralateral maxillary first molars underwent no treatment and served as normal control.


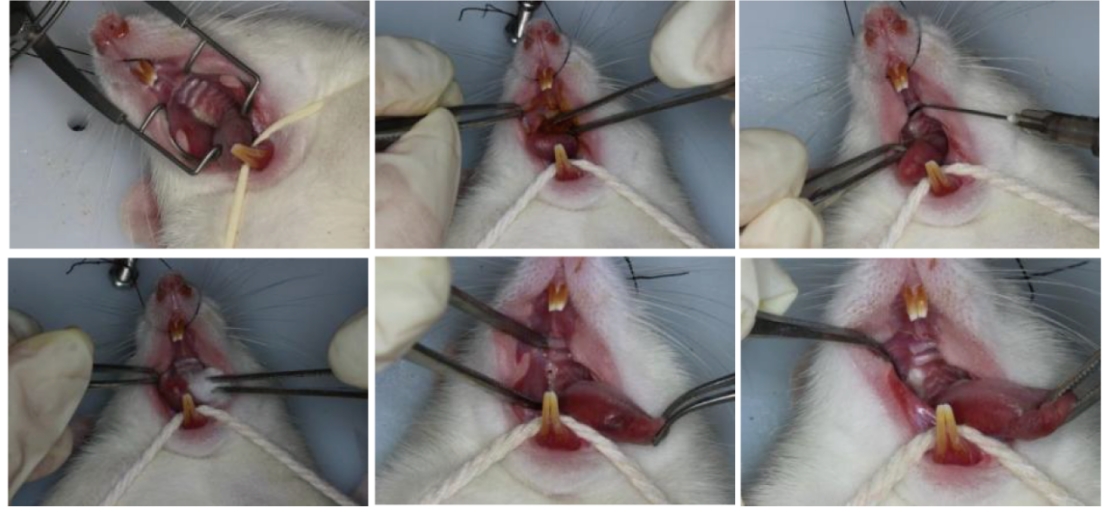


Figure S1. The construction of pulpitis in rat maxillary first molars


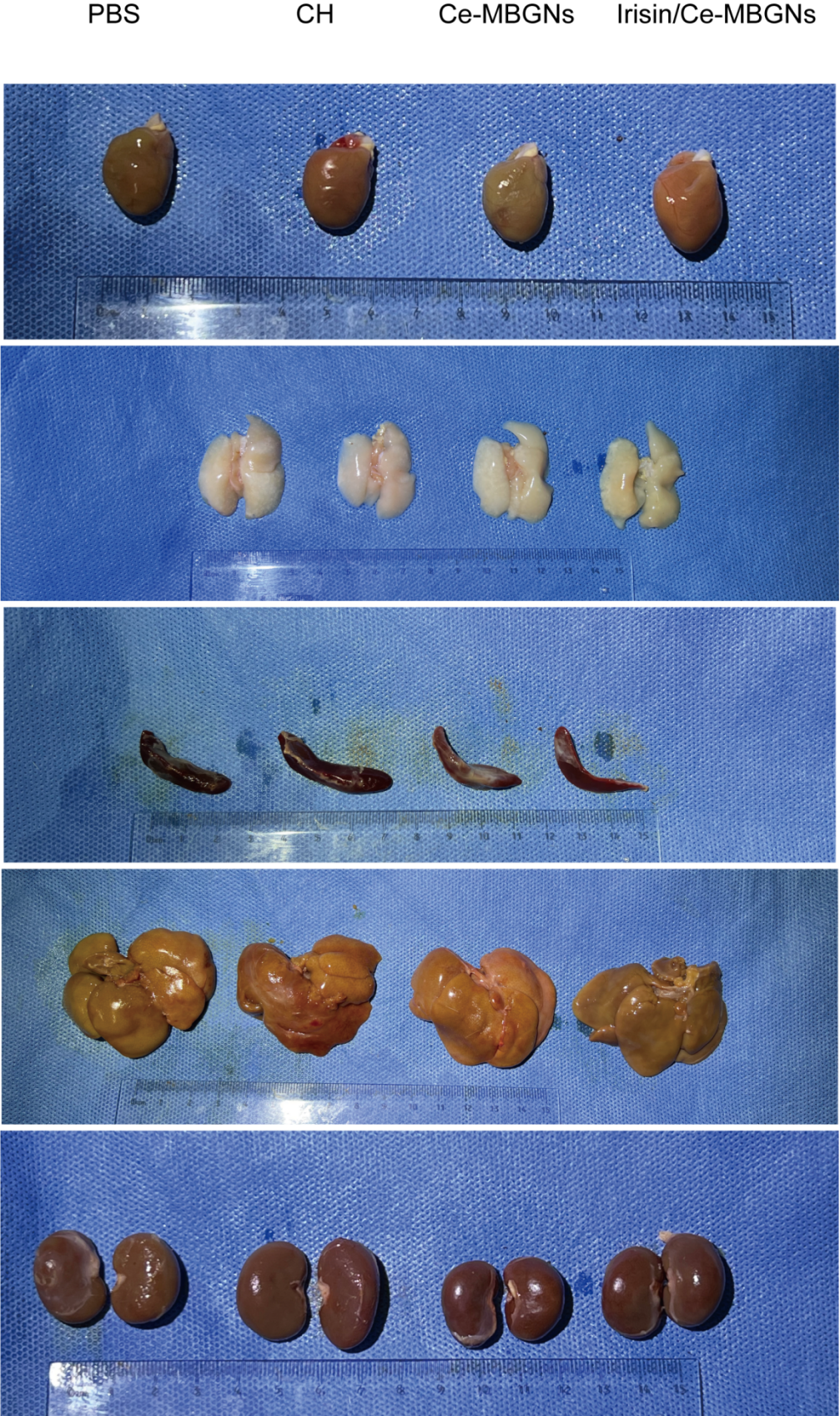


Figure S2. The size comparison of the heart, liver, spleen, lungs, and kidneys at the 8th week.

Table S1 Primers used for RT-qPCR.

| Genes | Primer sequences  Human species |
| --- | --- |
| Oct-4, octamer-binding transcription factor | Forward: 5′-TGAGAGGCAACCTGGAGAAT-3′  Reverse: 5′-AACCACACTCGGACCACATC-3′ |
| Sox2, SRY-Box Transcription Factor 2 | Forward: 5′-GGCAGAGAAGAGAGTGTTTGC-3′  Reverse: 5′-GCCGCCGATGATTGTTATT-3′ |
| Nanog, nanog homeobox | Forward: 5′-AAGGTCCCGGTCAAGAAACAG-3′  Reverse: 5′-CTTCTGCGTCACACCATTGC-3′ |
| ALP, alkaline phosphatase | Forward: 5′-AACATCAGGGACATTGACGTG-3′  Reverse: 5′-GTATCTCGGTTTGAAGCTCTTCC-3′ |
| DSPP, dentine sialophosphoprotein | Forward: 5′-GGGAATAGAAATCAAGGGTC-3′  Reverse: 5′-CAAGATCATTCCATGTTGTCC-3′ |
| DMP-1, dentin matrix acidic phosphoprotein 1 | Forward: 5′-GGTATCACACCCAACTATGAAGATCA-3′  Reverse: 5′-CCATCATACTGAGCAGCAAAGTTC-3′ |
| OCN, osteocalcin | Forward: 5′-CTCACACTCCTCGCCCTATTG-3′  Reverse: 5′-GCCTGGGTCTCTTCACTACC-3′ |
| BMP2, bone morphogenetic protein 2 | Forward: 5′-ACCCGCTGTCTTCTAGCGT-3′  Reverse: 5′-TTTCAGGCCGAACATGCTGAG-3′ |
| OPN, osteopontin | Forward: 5′-CTCCATTGACTCGAACGACTC-3′  Reverse: 5′-CAGGTCTGCGAAACTTCTTAGAT-3′ |
| Osterix | Forward: 5′-CCTCTGCGGGACTCAACAAC-3′  Reverse: 5′-AGCCCATTAGTGCTTGTAAAGG-3′ |
| RUNX2, runt-related transcription factor 2 | Forward: 5′-TGGTTACTGTCATGGCGGGTA-3′  Reverse: 5′-TCTCAGATCGTTGAACCTTGCTA-3′ |
| COL1A1, collagen type I alpha 1 chain | Forward: 5′-GAGGGCCAAGACGAAGACATC-3′  Reverse: 5′-CAGATCACGTCATCGCACAAC-3′ |
| IL-1Ra, interleukin-1 receptor antagonist | Forward: 5′-TTCCTGTTCCATTCAGAGACGAT-3′  Reverse: 5′-AATTGACATTTGGTCCTTGCAA-3′ |
| IL-1R1, interleukin-1 receptor type 1 | Forward: 5′-ATGAAATTGATGTTCGTCCCTGT-3′  Reverse: 5′-ACCACGCAATAGTAATGTCCTG-3′ |
| IL-1R2, interleukin-1 receptor type 2 | Forward: 5′-TCCTGCCGTTCATCTCATACC-3′  Reverse: 5′-CATCGTGTACGAGTAAGTGAGTG-3′ |
| MMP9, matrix metalloproteinase 9 | Forward: 5′-GGGACGCAGACATCGTCATC-3′  Reverse: 5′-TCGTCATCGTCGAAATGGGC-3′ |
| CTR, calcitonin receptor | Forward: 5′-CCTATCCAACAATAGAGCCCAAG-3′  Reverse: 5′-TGCATTCGGTCATAGCATTTGTA-3′ |
| CTSK, cathepsin K | Forward: 5′-ACACCCACTGGGAGCTATG-3′  Reverse: 5′-GACAGGGGTACTTTGAGTCCA-3′ |
| VEGF, vascular endothelial growth factor | Forward: 5′-CTGAACGATGAAGCCCTGGAG-3′  Reverse: 5′-TGGTGAGGTTTGATCCGCAT-3′ |
| bFGF, basic fibroblast growth factor | Forward: 5′-CATACAGCAGCAGCCTAGCAACTC-3′  Reverse: 5′-GTATTCGGCAACAGCACACAAATCC-3′ |
| ANG-1, angiopoietin-1 | Forward: 5′-GAGGTCAGAAGAAAGGAGCAA-3′  Reverse: 5′-AGAATGGCAGCGAGGAAAG-3′ |
| ANG-2, angiotensin-2 | Forward: 5′-GGGAAGGGAATGAGGCTTAC-3′  Reverse: 5′-GGTTGGCTGATGCTGCTTAT-3′ |
| GAPDH, glyceraldehyde-3-phosphate dehydrogenase | Forward: 5′-CAAGGTCATCCATGACAACTTTG-3′  Reverse: 5′-GTCCACCACCCTGTTGCTGTAG-3′ |

Table S2 Primers used for RT-qPCR.

| Genes | Primer sequences  Mouse species |
| --- | --- |
| IL-1β, interleukin-1β | Forward: 5′-ATGATGGCTTATTACAGTGGCAA-3′    Reverse: 5′-GTCGGAGATTCGTAGCTGGA-3′ |
| iNOS, inducible nitric oxide synthase | Forword: 5′-AGGGACAAGCCTACCCCTC-3′  Reverse: 5′-CTCATCTCCCGTCAGTTGGT-3′ |
| CD86 | Forward: 5′-TCTCCAACAGCCTCTCTCTTT-3′  Reverse: 5′-GAAACGGAGTCAATGAAGATT-3′ |
| CD206 | Forward: 5′-CTCTGTTCAGCTATTGGACGC-3′  Reverse: 5′-CGGAATTTCTGGGATTCAGCTTC-3 |
| IL-10, interleukin-10 | Forward: 5′-TCAAGGCGCATGTGAACTCC-3′  Reverse: 5′-GATGTCAAACTCACTCATGGCT-3′ |
| Arg1, arginase-1 | Forword: 5′-TGGACAGACTAGGAATTGGCA-3′  Reverse: 5′-CCAGTCCGTCAACATCAAAACT-3′ |
| TNF-α, tumour necrosis factor-alpha | Forward: 5′-CCTCTCTCTAATCAGCCCTCTG-3′  Reverse: 5′-GAGGACCTGGGAGTAGATGAG-3′ |
| IL-6, interleukin-6 | Forward: 5′-CTGCAAGAGACTTCCATCCAG-3′    Reverse: 5’-AGTGGTATAGACAGGTCTGTTGG-3′ |
| CCL5, chemokine (C-C motif) ligand 5 | Forward: 5′-CCAGCAGTCGTCTTTGTCAC-3′  Reverse: 5′-CTCTGGGTTGGCACACACTT-3′ |
| TGF-β1, transforming growth factor-beta | Forward: 5′-GGCCAGATCCTGTCCAAGC-3′  Reverse: 5′-GTGGGTTTCCACCATTAGCAC-3′ |
| GAPDH, glyceraldehyde-3-phosphate dehydrogenase | Forward: 5′-CCGAGACCAACCGAGTCATTTA-3′  Reverse: 5′- GATGGTGATGGGTTTCCCGT-3′ |

Table S3 Pathological evaluation and scoring of pulpal inflammation.

| Score | Inflammatory reaction |
| --- | --- |
| Score 1: Inflammatory cellular response of the coronal pulp | 1 point: Absence or scattered inflammatory cell infiltration in tissues  close to the pulp trauma site  2 points: Inflammatory cell infiltration in part of the pulp  3 points: Inflammatory cell infiltration in most of the pulp  4 points: all coronal pulp is infiltrated or necrotic |
| Score 2: Morphology of pulp tissue | 1 point: vasodilatory congestive changes only  2 points: disorganisation of the adult dentin cell layer  3 points: disorganisation of pulp tissue morphology  4 points: pulp necrosis |
| Score 3: Extent of pulp necrosis | 1 point: none or limited to upper third  2 points: limited to the middle third  3 points: more than middle third  4 points: complete necrosis |

Table S4 List of key findings and comparisons between Irisin/Ce-MBGNs and other VPT agents.

| Performance | Irisin/Ce-MBGN | MTA | Biodentine | IRoot BP |
| --- | --- | --- | --- | --- |
| Antioxidant | [High-performance SOD mimetic enzyme](#_edn1)^[3]^ | None | [Weak (calcium ion release related)](#_edn2)^[4]^ | None |
| Degradation | No significant degradation | [Non-degradable](#_edn3)^[5]^ | Non-degradable | Non-degradable |
| Hydrophilic/Hydrophobic | Hydrophilic | Hydrophilic (poor) | Hydrophilic | Hydrophilic |
| Anti-inflammatory | Significant (through multiple pathways) | Unstabitily^[6]^ | Medium (regulates macrophage polarization^[7, 8]^; significant early inflammatory response) | Inhibiting inflammation in a relatively short period of time^[9]^ |
| Quality of dentin bridge | Regular | Dense but with fractures; Slow speed | Dense and uniform | Thin and discontinuous |
| Long-term Effects | Long-term effective action (reducing the production of inflammatory factors in healthy pulp cells) | Long-term effective action (Inducing inflammation and even inhibiting mineralization^[10, 11]^ | Few relatively long-term clinical studies | |

**References**

[1] Arany, P. R., Cho, A., Hunt, T. D., et al., Photoactivation of endogenous latent transforming growth factor-β1 directs dental stem cell differentiation for regeneration, 2014, Sci Transl Med, 6, 238ra69, 10.1126/scitranslmed.3008234.

[2] Peng, X., Han, S., Wang, K., Ding, L., Liu, Z., Zhang, L., Evaluating the potential of an amelogenin-derived peptide in tertiary dentin formation, 2021, Regen Biomater, 8, rbab004, 10.1093/rb/rbab004.

[3] Yang, B., Chen, Y., Shi, J., Reactive Oxygen Species (ROS)-Based Nanomedicine, 2019, Chemical Reviews, 119, 4881, 10.1021/acs.chemrev.8b00626.

[4] Bossù, M., Mancini, P., Bruni, E., et al., Biocompatibility and Antibiofilm Properties of Calcium Silicate-Based Cements: An In Vitro Evaluation and Report of Two Clinical Cases, 2021, Biology (Basel), 10, 10.3390/biology10060470.

[5] Parirokh, M., Torabinejad, M., Mineral trioxide aggregate: a comprehensive literature review--Part I: chemical, physical, and antibacterial properties, 2010, J Endod, 36, 16, 10.1016/j.joen.2009.09.006.

[6] Silva, R. A. B., Gaton-Hernandez, P., Pucinelli, C. M., et al., Subcutaneous tissue reaction and gene expression of inflammatory markers after Biodentine and MTA implantation, 2022, Braz Dent J, 33, 41, 10.1590/0103-6440202203562.

[7] Eraković, M., Duka, M., Bekić, M., et al., Anti-inflammatory and immunomodulatory effects of Biodentine on human periapical lesion cells in culture, 2020, Int Endod J, 53, 1398, 10.1111/iej.13351.

[8] Abuarqoub, D., Aslam, N., Zaza, R., et al., The Immunomodulatory and Regenerative Effect of Biodentine™ on Human THP-1 Cells and Dental Pulp Stem Cells: In Vitro Study, 2022, Biomed Res Int, 2022, 2656784, 10.1155/2022/2656784.

[9] Zeng, Q., Zhou, C., Li, M., Qiu, Y., Wei, X., Liu, H., Concentrated growth factor combined with iRoot BP Plus promotes inflamed pulp repair: an in vitro and in vivo study, 2023, BMC Oral Health, 23, 225, 10.1186/s12903-023-02903-5.

[10] Chen, J., Qin, X., Zhong, S., Chen, S., Su, W., Liu, Y., Characterization of Curcumin/Cyclodextrin Polymer Inclusion Complex and Investigation on Its Antioxidant and Antiproliferative Activities, 2018, Molecules, 23, 10.3390/molecules23051179.

[11] Wang, M. C., Chang, K. W., Lin, S. C., Hung, P. S., Biodentine but not MTA induce DSPP expression of dental pulp cells with different severity of LPS-induced inflammation, 2023, Clin Oral Investig, 27, 1207, 10.1007/s00784-022-04734-0.
